# Recent advancements in zero- to three-dimensional carbon networks with a two-dimensional electrode material for high-performance supercapacitors

**DOI:** 10.1039/d3na00094j

**Published:** 2023-05-01

**Authors:** Niraj Kumar, Sudip Ghosh, Dinbandhu Thakur, Chuan-Pei Lee, Prasanta Kumar Sahoo

**Affiliations:** a Sustainable Energy Laboratory, Department of Metallurgical and Materials Engineering, Defence Institute of Advanced Technology (DIAT) Pune Maharashtra 411025 India; b Department of Chemistry, Siksha ‘O’ Anusandhan, Deemed to be University Bhubaneswar Odisha India; c Department of Metallurgical Engineering and Materials Science, Indian Institute of Technology Bombay Mumbai-400076 India; d Department of Applied Physics and Chemistry, University of Taipei Taipei 10048 Taiwan cplee@utaipei.edu.tw; e Department of Mechanical Engineering, Siksha ‘O’ Anusandhan Deemed to Be University Bhubaneswar 751030 India sahoo.barc@gmail.com

## Abstract

Supercapacitors have gained significant attention owing to their exceptional performance in terms of energy density and power density, making them suitable for various applications, such as mobile devices, electric vehicles, and renewable energy storage systems. This review focuses on recent advancements in the utilization of 0-dimensional to 3-dimensional carbon network materials as electrode materials for high-performance supercapacitor devices. This study aims to provide a comprehensive evaluation of the potential of carbon-based materials in enhancing the electrochemical performance of supercapacitors. The combination of these materials with other cutting-edge materials, such as Transition Metal Dichalcogenides (TMDs), MXenes, Layered Double Hydroxides (LDHs), graphitic carbon nitride (g-C_3_N_4_), Metal–Organic Frameworks (MOFs), Black Phosphorus (BP), and perovskite nanoarchitectures, has been extensively studied to achieve a wide operating potential window. The combination of these materials synchronizes their different charge-storage mechanisms to attain practical and realistic applications. The findings of this review indicate that hybrid composite electrodes with 3D structures exhibit the best potential in terms of overall electrochemical performance. However, this field faces several challenges and promising research directions. This study aimed to highlight these challenges and provide insights into the potential of carbon-based materials in supercapacitor applications.

## Abstract 2

Supercapacitors have gained significant attention owing to their exceptional performance in various applications, such as mobile devices, electric vehicles, and renewable energy storage systems.

## Graphical abstract

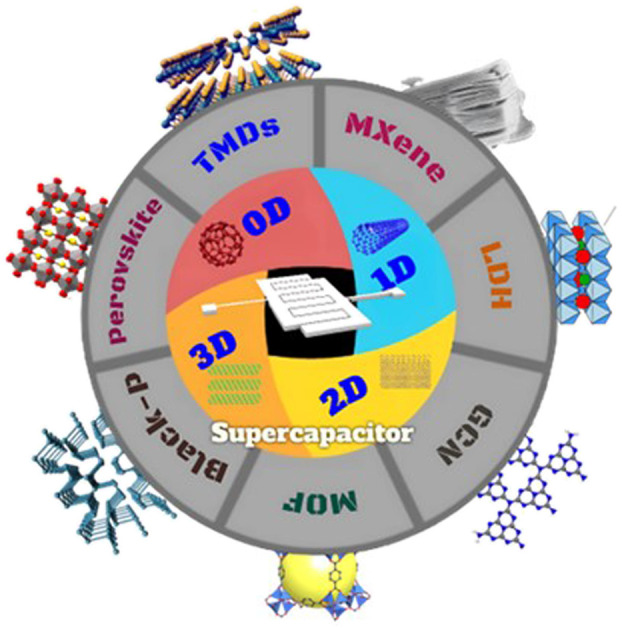

## 1. Introduction

The rising demand for clean and reliable energy sources, coupled with environmental concerns associated with traditional energy storage methods, has brought about the need for supercapacitors.^1,2^ The energy storage crisis has become an increasing concern, with the limitations of conventional energy storage methods, such as batteries, hindering their ability to efficiently store and discharge energy.^3–5^ The limited capacity and short lifespan of traditional energy storage methods not only affect their effectiveness but also contribute to environmental concerns such as waste disposal and finite resource depletion.^6^ Supercapacitors, with their ability to store and discharge energy quickly and efficiently, present a promising solution to the energy-storage crisis. Their high energy density, long lifespan, and eco-friendliness make them attractive options for meeting the growing demand for clean and dependable energy sources.^7^ Conventional energy storage methods, such as batteries, have been widely used in various applications including consumer electronics and electric vehicles. However, they have certain limitations that affect their efficiency in storing and discharging energy as required. One significant limitation of batteries is their limited capacity, which determines the amount of energy they can store at any given time. This limited capacity means that the amount of energy that can be stored is often insufficient for high-energy-demand applications, such as electric vehicles, which require larger amounts of energy for longer periods. In addition, batteries have a relatively short lifespan, which means that they require frequent replacement, leading to additional costs and waste. These limitations hinder the ability of conventional energy storage methods to efficiently store and discharge energy, thereby limiting their effectiveness and usefulness in many applications.^8^ Their exceptional capabilities position supercapacitors as potential major players in the resolution of the energy storage crisis and the realization of a more sustainable energy future. The electrodes in supercapacitors can be composed of a range of materials, including zero-dimensional (0D), one-dimensional (1D), two-dimensional (2D), and three-dimensional (3D) carbon-based materials. 0D carbon-based materials refer to activated carbon, which has a porous structure and a substantial surface area.^9,10^ The high mechanical strength and exceptional electrical conductivity of single-walled carbon nanotubes characterize 1D carbon-based materials.^11,12^ 2D carbon-based materials, such as graphene sheets, exhibit a high surface area and commendable electrical conductivity.^13–15^ 3D carbon-based materials are represented by carbon aerogels, which possess a high degree of porosity and extensive surface area.^16,17^ An activated carbon supercapacitor, which provides high capacitance and stability, is an example of a supercapacitor that employs zero-dimensional (0D) carbon-based materials.

2D electrode materials as electrode materials in supercapacitors are superior to traditional electrode materials owing to several advantageous properties. First, the high surface area of 2D materials results in a high capacitance and improved energy storage capacity.^18,19^ Second, the good electrical conductivity of 2D materials enables fast charge and discharge times, making them particularly suitable for high-power applications such as electric vehicles and renewable energy systems, where quick energy storage and release is necessary.^20^ Furthermore, 2D materials possess excellent mechanical strength, allowing them to endure high stress and repeated charge–discharge cycles.^21^ Additionally, the long-term performance of 2D materials is relatively stable compared with that of other electrode materials, thereby reducing the risk of degradation over time. These properties make 2D materials a superior option for use as electrode materials in supercapacitors. Zhang *et al.*^6^ summarized the recent progress in the preparation methods of biomass-based porous graphitic carbon (BPGC) as an electrode material in supercapacitors and its optimization and restructuring from 0D to 3D, while discussing its structure–performance correlation and challenges and opportunities for future development. This review helps researchers choose appropriate methods for constructing BPGC and opens up various directions for optimizing ion and electron transport for high-performance energy storage devices. Kumar *et al.*^3^ provided a comprehensive overview of the current state of the art in the fabrication and electrochemical performance of advanced electrode materials for supercapacitors, including various carbon nanomaterials with different dimensions and emerging fabrication technologies. They highlighted single atom, dual atom-doped and composites with carbon nanomaterials enhances the electrochemical performance of electrode materials and discussed the potential of supercapacitors to fill this technology gap. This article also highlights the challenges and promising research directions in this field. Benoy *et al.*^21^ provided a critical review of the progress and status of hybrid supercapacitor-battery energy storage devices, focusing on the use of porous and graphene-based carbon electrode materials and their electrochemical properties for potential applications in various sectors, particularly hybrid energy vehicles. The review of Zhong *et al.*^22^ highlights the current research progress in supercapacitors and the different carbon nanomaterials used as electrodes as well as the impact of multilevel composite structures on high-performance supercapacitors. This provides an insight into the development of high-energy supercapacitors.

**Fig. 1:**
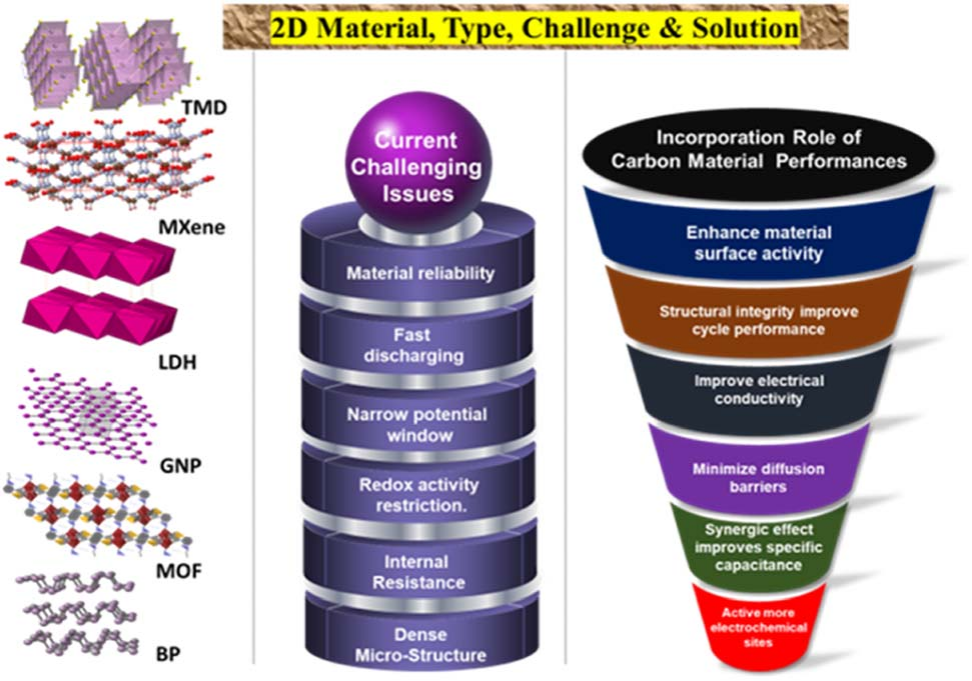
A schematic of 2D materials with the type, current challenges, and solutions.

Advancements in the utilization of carbon-based materials as electrode materials for supercapacitors have progressed from 0D to three-dimensional 3D structures, with (2D) materials exhibiting potential for high-performance applications. The evolution of activated carbon (0D) to single-walled carbon nanotubes (1D), and then to graphene sheets (2D), and finally to carbon aerogels (3D), has led to an increase in capacitance and stability. In particular, 2D materials, such as graphene, are deemed suitable for high-power applications owing to their exceptional surface areas and electrical conductivity. Ongoing research in this domain aims to enhance the production methods and long-term stability of 2D carbon materials, with the ultimate goal of realizing more cost-effective and efficient energy storage solutions. Fig. 1 shows a schematic representation of various types of 2D materials, highlighting their current challenges and possible solutions to overcome them.

This review paper delves into the latest developments in the utilization of 0-dimensional to 3-dimensional carbon network materials as electrode materials for high-performance supercapacitors. This study focuses on the integration of diverse carbon materials such as 0D, 1D, 2D, and 3D carbon materials with other state-of-the-art materials such as Transition Metal Dichalcogenides (TMDs), MXenes, Layered Double Hydroxides (LDHs), graphitic carbon nitride (g-C_3_N_4_), Metal–Organic Frameworks (MOFs), Black Phosphorus (BP), and perovskite. This study conducted a comprehensive evaluation of the potential of these combinations to enhance the performance of supercapacitors, thus making them even more efficient. Finally, this paper highlights the future prospects of these combinations and the likelihood of further advancements in the field of high-performance supercapacitors.

## 2. Carbon materials

### 2.1 Zero dimensional carbon materials

Carbon materials with dimensions of zero are spherical and have an aspect ratio of ∼1. They mainly include mesoporous carbon, activated carbon (AC), carbon black, and carbon nanospheres. Zero-dimensional carbon materials such as carbon dots, carbon quantum dots, and graphene quantum dots have unique physical and chemical properties that make them promising candidates for supercapacitor applications. They have high surface areas, can store charges at the nanoscale level, and their surface chemistry is easily tunable through chemical modifications to enhance their electrochemical performance.^23,24^ They have a large specific surface area (hundreds to thousands of m^2^ g^−1^) as well as pore sizes and dispersion that can be adjusted.^25^ Zero-dimensional carbon particles can be produced by physical activation and chemical activation processes. Physically activation process is performed at a temperature of 700–1200 °C in the presence of different oxidizing gases, such as CO_2_, H_2_O, and air whereas the chemical activation process is carried out at a temperature of 600–800 °C in the presence of KOH, H_3_PO_4_, and ZnCl_2_.^26^ The large surface area, high electrical stability, and low cost make AC a promising material that can be used for supercapacitors. Basic materials like coal, wood, *etc.*, that have plentiful carbon are either physically or chemically activated to obtain AC. In the lack of atmospheric air, those basic materials are heated to an extremely high temperature of 1200 °C during physical activation. Similarly, in the presence of activity agents like zinc chloride, sodium hydroxide, phosphoric acid, *etc.*, those basic materials are heated at lower temperatures of 400–700 °C during the chemical activation process.^9^ Yue *et al.*^9^ reviewed the binary and ternary composites of carbon material/MnO_2_ for high-performance supercapacitor electrode materials, discussing their mechanisms and practical applications and analyzing their characteristics. They conclude that multi-component composites, especially biomass activated carbon/low-dimensional carbon materials (1D, 2D)/MnO_2_), will be promising materials for supercapacitors, and that new technologies such as graft oxidation and 3D printing will be the future trend in their preparation. The challenges and development trends of these composites are also discussed. Peçenek *et al.*^19^ highlighted the potential of carbon-based MnO_2_ composite electrodes for energy storage applications, with high conductivity and a large surface area resulting from the favorable interaction between MnO_2_ and nanoscale materials. Nitrogen-doped MnO_2_ composite supercapacitor electrodes with high specific capacitance and good cycle performance have been demonstrated, with the highest specific capacitance (457 F g^−1^ at 1 A g^−1^) achieved by a MXene-based MnO_2_ composite. Liu *et al.*^21^ showed the preparation of MLCM from ionic liquid-lignin solution, which offers a green, facile and sustainable strategy for high-performance carbon electrode materials, specifically from renewable lignin. They prepared nitrogen and phosphorus dual-doped alkali lignin-based carbon microspheres and used as supercapacitor electrode materials. They showed superior electrochemical performance with the highest specific capacitance of 338.2 F g^−1^ and maintained excellent cycle stability after 5000 charging/discharging cycles.

### 2.2 One dimensional carbon materials

One dimensional carbon particles have a long one-dimensional nanostructure; therefore, one dimensional carbon particles are widely used for supercapacitors.^25^ For the manufacturing of one-dimensional carbon materials, fibrous biomass such as wood, cotton, and flax is used, with cellulose being the essential element of biomass.^27^ One-dimensional carbon materials, such as carbon nanotubes, carbon nanofibers, and graphene nanoribbons, have unique structural and electronic properties that make them a promising choice as electrode materials for supercapacitors. Their high aspect ratio and electronic properties result in a large surface area and faster charge and discharge rates, enabling a higher capacitance. In addition, their high mechanical strength makes them good candidates for applications that require high-stress conditions.^28,29^ For example, CNTs are the most typical one-dimensional carbon particles. CNTs are classified into single-walled CNTs and multi-walled CNTs, prepared by the arc discharge process, pyrolysis of hydrocarbons, and CVD method.^30–32^ The abundant surface area, light weight, electrical conductivity properties, and intrinsic flexibility of carbon nanotubes make them the most commonly used supercapacitor electrode material.^33^ Xiong *et al.* used electrophoretic deposition followed by the chemical vapor deposition method to fabricate a rGO/CNT composite on CF. This combination reported a *C*_sp_ of 203 F g^−1^, 4 times higher than that of pure CF.^34^ CNFs represent another typical one-dimensional carbon material that may be produced using the chemical vapor method.^35–38^ Recently, Selvaraj *et al.*^10^ have developed a low-cost method for fabricating high-performance supercapacitors using carbon nanofibers, graphene oxide sheets, and manganese dioxide, resulting in a specific capacitance of 271.4 F g^−1^ and energy density of 17.3 W h kg^−1^ for CNF/MnO_2_ and 251.5 F g^−1^ at 0.5 A g^−1^ for rGO/CNF. This result has potential to be a high-performance and cost-effective energy storage solution for the future. Zhang *et al.*^39^ presented a versatile design for fabricating MOF-based heterostructures as energy storage electrodes, offering improved electrochemical performance through increased interfacial active sites and better reversible redox reaction kinetics. They fabricated a Co-carbonate hydroxide@Ni-metal–organic framework (Co–CH@Ni-MOF) composite with a super-uniform core–shell heterostructure and demonstrated to have a high specific capacity of 173.1 mA h g^−1^ and excellent cycling stability. Shivakumar *et al.*^40^ showed that the one-dimensional hollow cuboid-like architecture of CFMO micro-/nanostructures offers improved electrochemical performance and stability, making it a promising material for hybrid supercapacitors in future energy storage devices. One-dimensional hollow FeMoO_4_ (CFMO) micro-/nanostructures were fabricated through a hydrothermal approach and found to exhibit superior electrochemical performance compared to non-calcined FeMoO_4_ (AFMO) materials. CFMO showed a maximum specific capacitance of 493 F g^−1^ and the resulting hybrid supercapacitor (CFMO//AC) had a maximum energy density of 29.89 W h kg^−1^ and a power density of 1001.58 W kg^−1^ with 89.62% capacitance retention after 10 000 cycles.

### 2.3 Two dimensional carbon materials

2-D carbon materials are sheet-like structure particles such as reduced graphene oxide, graphene, graphene oxide, nano-films and nano-plates. Two-dimensional carbon materials, such as graphene and graphene oxide, are highly sought-after electrode materials for supercapacitors because of their unique two-dimensional structure and excellent electrical conductivity. The high surface area and abundance of edges and defects make them highly suitable for charge storage, enabling faster charge and discharge rates and higher capacitance.^41^ Furthermore, their surface chemistry can be easily modified to enhance their electrochemical performance.^28^ Graphene is a one atomic thick sheet that possesses some important properties like exceptional electrical and thermal conductivity, high tunable surface area (2675 m^2^ g^−1^), structural flexibility, short diffusion distance, strong mechanical strength, good chemical stability, and good chemical stability with a wide potential window.^42–45^ A *C*_sp_ of 75 F g^−1^ with an energy density of 31.9 W h kg^−1^ was reported by Dubey *et al.* utilizing a graphene-based supercapacitor device having an ionic liquid electrolyte while with organic and aqueous electrolytes, it yields a *C*_sp_ value of 99 F g^−1^ and 135 F g^−1^, respectively.^30^ Several methods for the preparation of graphene are already reported, and out of them, the “Scotch-tape” process is mainly used. Meanwhile for the study basis, several methods like chemical vapor deposition, arc discharge, exfoliation, *etc.*, are used. Activated Carbon Nanofiber (ACNF) mats were compared by Singh *et al.*^30^ with Activated Carbon (AC) and Crushed Carbon Nanofibers (CA-CNF) for use as electrode materials in supercapacitors using various characterization techniques. ACNF showed the highest specific capacitance of 203.29 F g^−1^ compared to AC (106.17 F g^−1^) and CA-CNF (166.12 F g^−1^) and an all-solid-state supercapacitor device made with ACNF mats exhibited high energy density (65.52 W h kg^−1^) and power density (1036.27 W kg^−1^) with high coulombic efficiency (99.6%) after 10 000 cycles. MoS_2_/Mn-MOF/MWCNT composites were synthesized by Peng *et al.*^46^ and tested for supercapacitor performance, with the best result of 862.73 Fg^−1^ specific capacitance achieved at a mass ratio of 1 : 4 : 1 for MoS_2_ : Mn-MOF : MWCNT. The 2D based composite shows the best electrochemical performance among the tested materials and provides a promising method for the development of Mn-based supercapacitor materials. Ji *et al.*^47^ demonstrated a one-step calcination strategy to fabricate nickel sulfide and cobalt sulfide nanoparticles on ultrathin carbon two-dimensional nanosheets (Ni_3_S_2_/Co_9_S_8_/C) for use as a battery-type cathode in hybrid supercapacitors. The resulting Ni_3_S_2_/Co_9_S_8_/C cathode showed remarkable electrochemical features with a capacity of 1160 C g^−1^ (2320 F g^−1^) at 1 A g^−1^, good cycling capability, and a high energy density of 69.6 W h kg^−1^. This suggests great potential for practical application of Ni_3_S_2_/Co_9_S_8_/C ultrathin nanosheets.

### 2.4 Three dimensional carbon materials

In 3-D carbon materials, all the material dimensions remain outside the nanoscale (<100 nm). Graphite is a typical 3-D carbon material. The microstructure of electrode materials significantly impacts energy storage system performance.^26,48–50^ With the increase in dimensionality, the electrolytes come into contact with a more percentage of active sites, which improves the electrochemical performance of electrode materials. As a result, 3D layouts provide uninterrupted pathways for good electrolyte contact while simultaneously accelerating charge transfer by minimizing the diffusion path.^50–55^ Typically chemical vapor deposition, template, and hydrothermal methods create 3D carbon materials on a flexible surface like polymer surfaces or metal foam.^56–58^ A high graphitic porous biomass carbon (HGPBC) derived by Tan *et al.*^59^ from dandelion flower stems was synthesized using potassium ferrate as an activator and showed improved conductivity with a high specific capacitance of 309 F g^−1^ and energy density of 14.22 W h kg^−1^. The synthesis of HGPBC from a renewable source holds promise for low-cost, green production of advanced energy storage materials for supercapacitor applications in the future. 3-D activated carbon nanosheets modified with graphitized carbon dots (3-D ACNs/GCDs) by Li *et al.*^60^ have been proposed as a promising electrode material for supercapacitors. The product has a well 3-D porous structure and high specific surface area of 1328 m^2^ g^−1^. The capacitive performance of the 3-D ACNs/GCD electrode material is satisfactory with a high specific capacitance of 202.9 F g^−1^ at 1 A g^−1^ and good rate performance, as well as long cycle stability with 93.2% capacitance retention after 2000 cycles.

**Fig. 2:**
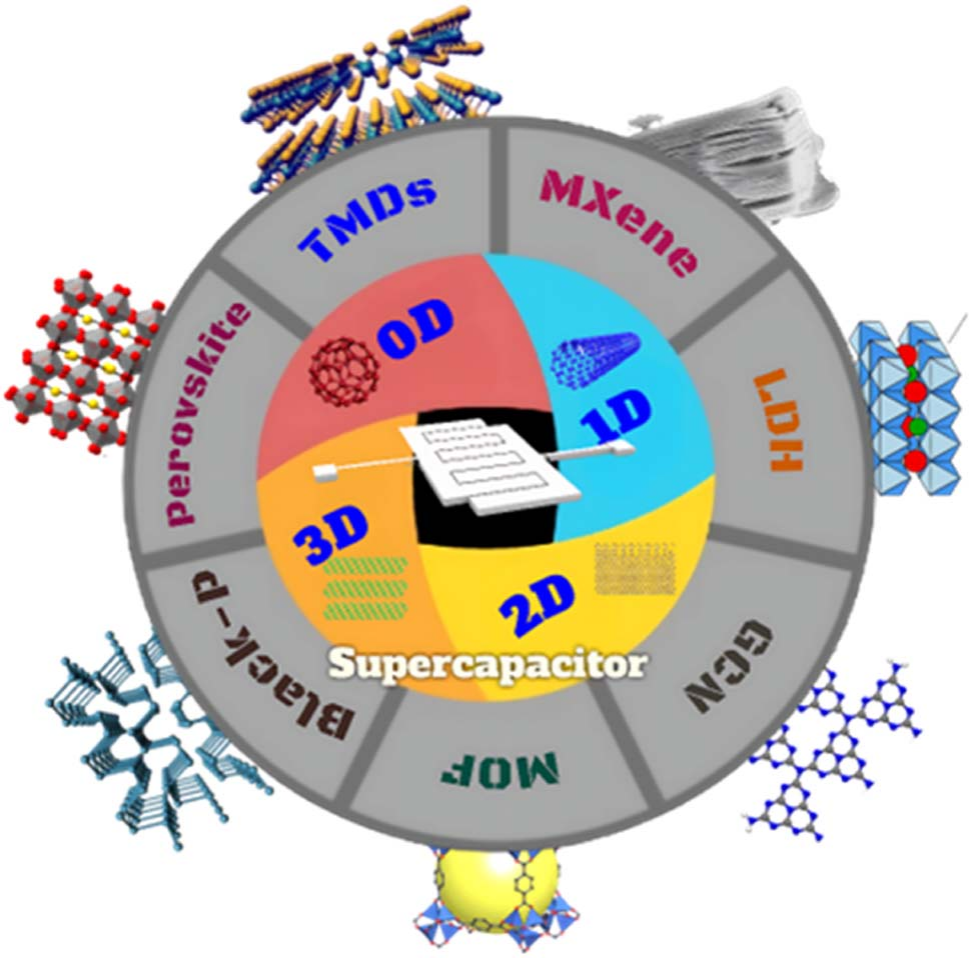
Schematic design of a 0D-3D carbon network with 2D electrode materials for supercapacitor applications using a rational approach.

Bi *et al.*^61^ developed a hierarchical porous carbon (HPC) and used it to create a MnO_2_@HPC composite as an electrode material for supercapacitors through a simple hydrothermal method. The MnO_2_@HPC-100 composite showed excellent performance with a high specific capacity of 368 F g^−1^, energy density of 88.2 W h kg^−1^, and stable capacity retention rate of 95% after 10 000 cycles, making it a promising material for energy storage applications. Fig. 2 shows a schematic design of a 3D carbon network integrated with 2D electrode materials for supercapacitor applications, which uses a rational approach.

## 3. Transition metal dichalcogenides (TMDs) in supercapacitors

**Fig. 3:**
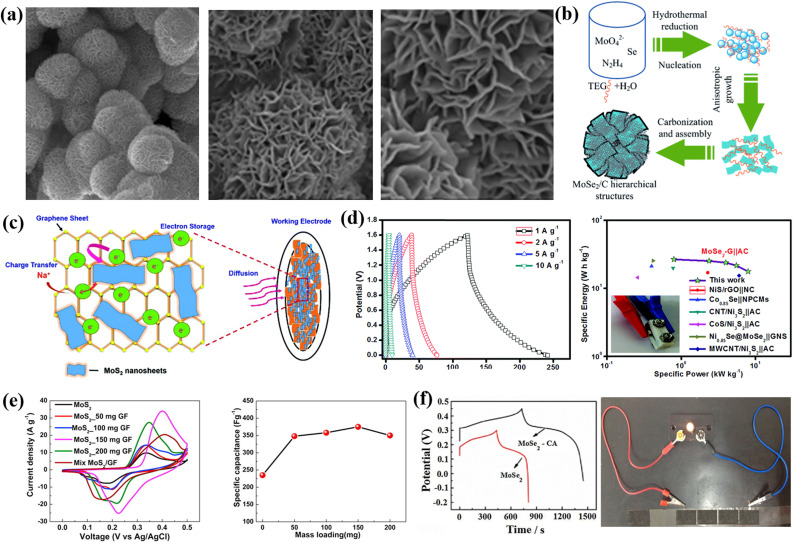
(a) Micrographs of f-MoS_2_ obtained using SEM. (b) An illustration of the formation of the hierarchical MoSe_2_/C hybrid. (c) A schematic showing the stability of MoS_2_ nanosheets on graphene. (d) The CD curves of the MoSe_2_/G nanohybrid at different current densities ranging from 1 to 10 A g^−1^, as well as a Ragone plot for the MoSe_2_/G‖AC ASC device, which also includes a photograph of the ASC device. (e) A comparison of CV curves and capacitance results, and (f) GCD curves and a demonstration of the LED.

TMDs are inorganic layered compounds with an X–M–X skeleton made up of chalcogens (X: Te, Se, S) and transition metals (M). TMDs have the generic formula MX_2_. M represents transition metals (W, Mo, *etc.*) and X represents chalcogens (S, Se, and Te). Basically, in TMDs, one metal atom (M) is sandwiched between two chalcogen (X) atoms.^62^ For example, molybdenum diselenide (MoSe_2_), molybdenum disulphide (MoS_2_), tungsten diselenide (WSe_2_), tungsten disulphide (WS_2_), *etc.*, are the most commonly used TMDs for supercapacitor electrode materials. Among the various TMDs, MoS_2_ is the most popular and studied TMDs. But regarding the supercapacitor application, MoSe_2_ is better than MoS_2_ due to its exceptional properties such as outstanding electrical conduction and lower size than MoS_2_.^63^ TMDs possess two types of phases: 1T phase and 2H phase, with the 1T phase being metallic and the 2H phase being semiconducting.^64^ TMDs have attracted a lot of attention as electrode materials because of their novel sheet-like morphology, abundant surface area, variable oxidation states, *etc.* The active edges in 2D TMDs allow us to combine them with other electrochemically active materials to form super active nanocomposites. TMDs have unique properties that allow them to store charge both electrostatically and *via* a faradaic mechanism.^65,66^ However, the poor 2H phase of TMDs hinders their real potential.^67^ The restacking of 2D TMDs also inhibits the large surface area which is available for electrolytes.^68,69^Fig. 3 shows a comprehensive schematic that includes a representation of synthesis routes and types of TMDs, their superconducting performance, a hybrid structure of 2D TMDs and graphene, and a crystalline structure of 1T TiS_2_. Fig. 3 encompasses a range of micrographs and illustrations that highlight the features of f-MoS_2_. (a) shows SEM micrographs of f-MoS_2_, (b) illustrates the formation process of the hierarchical MoSe_2_/C hybrid, (c) presents a schematic of the stability of MoS_2_ nanosheets on graphene, (d) displays the CD curves of the MoSe_2_/G nanohybrid at various current densities along with a Ragone plot for the MoSe_2_/G‖AC ASC device, (e) presents comparative CV curves and capacitance results, and (f) shows GCD curves and LED demonstration.

### 3.1 0D carbon-TMD composite electrode materials for supercapacitors

Sangeetha *et al.* described activated carbon/MoS_2_ composite synthesis for good performance ultracapacitors by a hydrothermal method. A different weight ratio (1 : 1, 1 : 2, 1 : 3, 2 : 1, and 3 : 1) of activated carbon/MoS_2_ was taken to fabricate the working electrode, and the total mass loading was maintained to be 2–5 mg cm^−2^. A symmetric supercapacitor was prepared by using two AC/MoS_2_ electrodes and showed a higher specific capacitance of 261 F g^−1^ at a scan rate of 2 mV s^−1^. A performance retention of about 89% over 5000 cycles was observed, which confirms its good cycling stability. Moreover, the nano-engineered material produced an energy density of 21 W h kg^−1^ and a power density of 225 W kg^−1^ for symmetric supercapacitors. Furthermore, they also constructed a hybrid supercapacitor with an AC anode and with an AC/MoS_2_ cathode. At 2 mV s^−1^, a *C*_sp_ of 193 F g^−1^ was observed. The hybrid supercapacitor's energy density was found to be 17 W h kg^−1^, and the power density was found to 200 W kg^−1^.^70^ Khawula *et al.* reported constructing a molybdenum disulfide modified carbon nanosphere (MoS_2_/CNS) nanoarchitecture as an electrode material for supercapacitors with two distinct morphologies (flower and spherical) by adopting a hydrothermal process. From the physical and chemical characteristics, they revealed that f-MoS_2_/CNS presented lattice development and a large surface range; on the other hand, s-MoS_2_/CNS showed lattice shrinkage and a reduced surface area. Moreover, f-MoS_2_/CNS exhibited an energy density of 26 W h kg^−1^, a specific capacitance of 231 F g^−1^, and a power density of 6443 W kg^−1^, while the *C*_sp_ of s-MoS_2_/CNS was 108 F g^−1^, with a power density of 3700 W kg^−1^ and energy density of 7.4 W h kg^−1^. The excellent electrochemical properties of the as-prepared composites are due to the sound structure of the composites and the synergetic effect of CNSs and MoS_2_ plates.^71^ Ma *et al.* adopted a simple hydrothermal approach to construct a 3D hierarchical flower-like MoSe_2_/C hybrid assembled with numerous few-layered nanosheets as the building blocks. Triethylene glycol acts as a source of carbon and a structure-directing substance. At 1 A g^−1^, the as-synthesized composite exhibited a remarkable capacitance performance of 878.6 F g^−1^, while bare MoSe_2_ displayed a capacitance performance of 436.7 F g^−1^. Moreover, the composite exhibited outstanding cycling performance, with 98% of the capacitance remaining after 2000 cycles. The high electrochemical performance is due to the hierarchical porous structure and the incorporation with the conductive carbon material, which offers a large surface area and promotes the charge transfer of carriers at the electrode/electrolyte interface.^72^

### 3.2 1D carbon-TMD composite electrode materials for supercapacitors

Tiwari *et al.* reported the decoration of MoS_2_@CNT nanostructures by utilizing a unique combination of CVD and PVD techniques, where the CNTs acted as templates for confining and directing the growth of MoS_2_ nanosheets to form a curved structure around them. Both faradaic and non-faradic processes are involved in the charge storage mechanism. At a scan rate of 5 mV s^−1^, the architecture exhibited a superior capacitance of 337 mF cm^−1^. Moreover, a remarkable performance retention of 97.6% over 2000 cycles has been reported. The composite electrode displayed a high volumetric capacitance of 2.9 F cm^−3^ and areal capacitance of 131 mF cm^−2^. The extraordinary electrochemical performance of the composite can be attributed to its open porous network, which provides short ionic and electron diffusion lengths and a large number of intercalation sites.^73^ Karade *et al.* utilized a scalable and simple two-step scheme to synthesize a MoSe_2_/multiwalled carbon nanotube (MoSe_2_/MWCNT) composite for supercapacitor applications, which exhibited an outstanding capacitance performance of 192 mA h g^−1^ specific capacity and 88% capacitance retention over 2000 cycles in an electrolyte of 1 M LiCl. In addition, the composite produced an energy density of 17.9 W h kg^−1^. Furthermore, an asymmetric supercapacitor system was built utilizing a pseudocapacitive material such as MnO_2_ as the positive electrode, which had a capacitance retention of 80% after 2000 consecutive cycles and a maximum *C*_sp_ of 112 F g^−1^ with an energy density of 35.6 W h kg^−1^. The large surface area of the MWCNT nanonetwork, which also provided a suitable conductive channel, was responsible for the remarkable performance of the composite. The interfacial conjugation and synergetic effect of MoSe_2_/MWCNTs correspondingly play a vital role in the composite's excellent performance.^74^ Hu *et al.* prepared an innovative supercapacitor electrode material in which WS_2_ nanoparticles were enclosed in amorphous carbon tubes (WS_2_NPs/CTs). In this study, WS_2_NPs were successfully grown on CTs *via* the carbothermal reduction of WS_4_^2−^ by pyrolyzing carbon from glucose. At 1 A g^−1^, the prepared composite exhibited a capacitance of 536 F g^−1^. In addition, it exhibited a capacitance of 337 F g^−1^ even at a high current density of 10 A g^−1^. At a high current density, it also shows 100% efficiency retention over 100 cycles and 60% after 500 cycles, resulting in outstanding cycling stability. The excellent supercapacitance is due to the small dimensions of WS_2_NPs and good electron transport between CTs. In addition, the low carbonization temperature of the composite makes the CTs amorphous and porous which allows the Li^+^ to move through freely.^75^

### 3.3 2D carbon-TMD composite electrode materials for supercapacitors

**Fig. 4:**
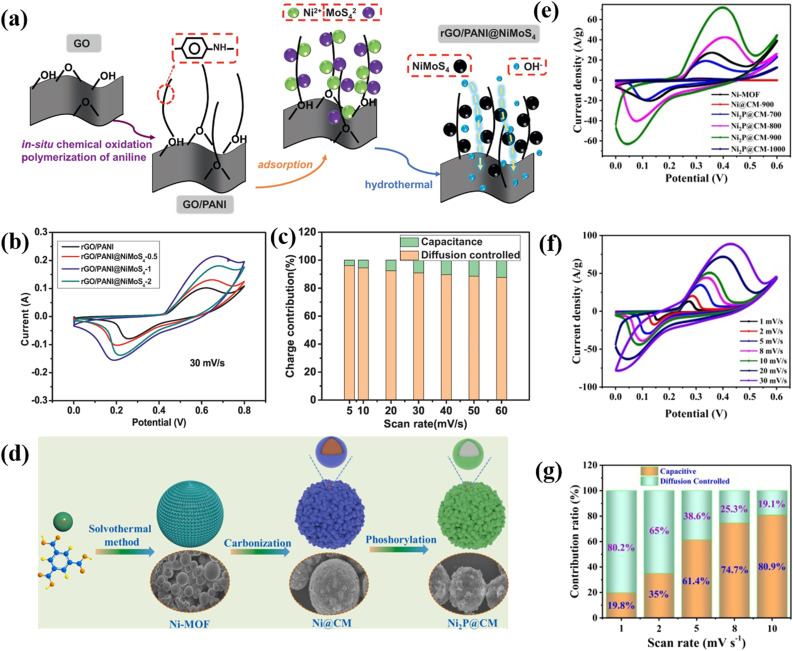
(a) Preparation process schematic of rGO/PANI@NiMoS_4_, (b) CV curves at varying scan rates, (c) charge involvement of rGO/PANI@NiMoS_4_, (d) preparation process schematic of Ni_2_P@CM, a nanoscale nickel phosphide encapsulated in a carbon microsphere, (e) comparison of CV curves of composites, (f) CV curves of Ni_2_P@CM-900 at scan rates ranging from 1 mV s^−1^ to 30 mV s^−1^, and (g) specific capacitance of composites.

Thangappan *et al.* decorated graphene with MoS_2_ nano-sheets for high performance supercapacitor applications by a facile one step preparation. At a current density of 0.1 A g^−1^, the as-prepared composite produced a supreme *C*_sp_ of 270 F g^−1^ in a neutral electrolyte (aqueous). It also delivers an energy density of 12.5 W h kg^−1^ as well as a high-power density of 2500 W kg^−1^. In addition, after 1000 consecutive cycles, the as-prepared composite still shows remarkable cycling stability. This higher specific capacitance value, high power density, high energy density, and excellent cycling stability are due to the interconnected conductive networks of the composites. Here graphene acts as an electronic conductive channel in the composite, which helps the fast transfer of electrons. The synergetic effect of pure graphene and MoS_2_ nanosheets plays an important role in the enhancement of the capacitive value in the composite.^76^ Kirubasankar *et al.* described the preparation of a MoSe_2_/graphene nanohybrid as a high-performance supercapacitor electrode material. To synthesize MoSe_2_, they used a simple and straightforward sonochemical method. They used a simple solvothermal approach to produce 2D MoSe_2_ engineered with graphene to enhance the capacitive properties. The as-prepared composite displays an excellent capacitance value of 945 F g^−1^ at 1 A g^−1^ current density. The high specific capacitance value of the composite is due to the presence of a large number of electrochemically active sites on the edges of MoSe_2_ nanosheets. Furthermore, they have constructed an unsymmetric ultracapacitor system (MoSe_2_/G/AC) which produced a *C*_sp_ of 75% at a current density of 1 A g^−1^. The as-synthesized asymmetric supercapacitor device also exhibits a power density of 0.8 kW kg^−1^ and an energy density of 26.6 W h kg^−1^ with a retention of 88% of its capacitance after 3000 cycles.^77^ Tu and coworkers reported using a facile molten salt technique to prepare a supercapacitor electrode material made of a two-dimensional hybrid WS_2_ and reduced graphene oxide nanostructure that exhibits a remarkable *C*_sp_ of 2508.07 F g^−1^ at a 1 mV s^−1^ scan rate. High cycling stability of 98.6% retention over 5000 cycles is also achieved. At a high-power density of 400 W kg^−1^, the as-prepared composite produced a good energy density of 28.33 W h kg^−1^. In addition, a coulombic productivity of about 100% for the complete investigation is also obtained for the composite. The synergetic effect of the extremely capacitive reduction–oxidation reaction of WS_2_ and excessive electron transmission capacity of rGO is the key factor for the highly enhanced electrochemical properties of the WS_2_/rGO composite.^78^ Xiong *et al.* demonstrated a ternary reduced graphene oxide (rGO)/polyaniline (PANI)@NiMoS_4_ composite with a high specific capacity of 194 mA h g^−1^ and a high energy density of 30.75 W h kg^−1^, as well as good cycle stability.^79^ The composite has potential for supercapacitor applications due to its high electrochemical performance. Fig. 4 illustrates the various aspects of the research study. (a) The preparation process schematic of rGO/PANI@NiMoS_4_ is shown to give an insight into how the material was synthesized. (b) CV curves at varying scan rates were analyzed to evaluate the electrochemical behavior of the material under different conditions. (c) The charge involvement of rGO/PANI@NiMoS_4_ was determined to understand its properties and the impact of the preparation process on them. (d) The preparation process schematic of Ni_2_P@CM, a nanoscale nickel phosphide encapsulated in a carbon microsphere, was presented to give an understanding of how it was synthesized. (e) The comparison of CV curves of different composites allowed for the evaluation of their performance and comparison to each other. (f) The CV curves of Ni_2_P@CM-900 were analyzed at scan rates ranging from 1 mV s^−1^ to 30 mV s^−1^ to understand its behavior at different scan rates. (g) Finally, the specific capacitance of the composites was determined to understand their energy storage capability.

### 3.4 3D carbon-TMD composite electrode materials for supercapacitors

Masikhwa and coworkers developed an asymmetric supercapacitor system with activated carbon and MoS_2_/graphene foam. To improve the capacitance performance of the composite, they used a hydrothermal technique to manufacture MoS_2_/graphene foam (GF) composites with various loadings of graphene foams. An asymmetric supercapacitor device was constructed by utilizing AC obtained from expanded graphite (AEG) and the MoS_2_/GF composite (150 mg mass loading) as the negative and positive electrodes in a 6 M KOH electrolyte and showed a maximum *C*_sp_ of 59 F g^−1^ at a current density of 1 A g^−1^ with a supreme energy density of 16 W h kg^−1^ and power density of 758 W kg^−1^. The composite additionally exhibits a performance retention of 95% over 2000 cycles.^80^ He and coworkers applied a simple process to prepare a novel 3D heterostructure of carbon aerogel nanospheres decorated with ultrathin MoSe_2_ nano-slices. At 1 A g^−1^ it exhibits a high specific capacity of 775.3 C g^−1^ and good cycle life of about 98% capacity retention after 1500 cycles in a 6 mol L^−1^ KOH electrolyte. Furthermore, it has been seen that the as-prepared composite displayed an increased energy density of 84.2 W h kg^−1^ as well as a power density of 5308 W kg^−1^. They used carbon aerogels for increasing capacitive performance because of their fascinating characteristics, such as good electroconductivity (100 s cm^−1^), high porosity, and large surface area (1100 m^2^ g^−1^).^81^ Shang *et al.* reported the preparation of interwoven WS_2_ nanofilms reinforced on carbon fibre cloth (WS_2_/CFC) by a facile solvothermal process which produced a *C*_sp_ of 399 F g^−1^ at 1 A g^−1^, which is superior to that of pure bulk WS_2_. The WS_2_/CFC composite also exhibits a high cycling stability of 99% capacitance retention after 500 cycles. The 3D framework of CFC provides a large surface to disperse WS_2_ nanoplates excellently. In addition, it also serves as an exceptional conduction substrate to boost electron transport frequency, which further improves the electrochemical performance of the WS_2_/CFC composite.^82^

Recent advancements in 2D thin transition metal dichalcogenides (TMDs) have been found to be promising for improving the energy storage techniques for supercapacitors. 2D TMDs have a thin structure similar to that of graphene, which allows for fast charge/discharge processes. They also have electrochemical behaviors that are both “faradaic” and “non-faradaic”, with higher adsorption energy and lower ion diffusion barriers, which improves energy, power, and cycling performances. However, energy storage research on 2D TMDs is still in its early stage and device demonstrations based on monolayered or thin-layered TMDs are limited. The main challenges include the fabrication of strictly monolayered TMDs with high yields on a large scale, the stabilization of thermally unstable 1T TMDs, and the poor electrical conductivity of stable 1H/2H TMDs. Future improvements may include the stabilization of 1T TMDs through chemical doping to improve the phase yield and electrochemical performance and the exploration of stable 2D ternary sulfides for energy storage.

## 4. MXenes for supercapacitors

MXenes, a group of two-dimensional transition metal layered carbonitrides and transition metal carbides, have attracted a lot of attention for the reason that they possess amazing chemical and physical properties.^83–85^ In addition, MXenes being safe with large interlayer spacing, outstanding biocompatibility and environmental flexibility have also attracted attention.^86^ The general formula of MXenes is M_*n*+1_X_*n*_T_*x*_. The M stands for a transition metal, X stands for carbon and/or nitrogen, and T stands for a surface termination group (such as –OH, –F, –O, *etc.*).^87^ In 2011, for the first time, a multilayer MXene (Ti_3_C_2_T_*x*_) was reported. Ti_3_C_2_T_*x*_ was obtained by etching an A layer from the MAX phase of Ti_3_AlC_2_.^88^ A MAX layer consists of hexagonally layered ternary transition metal carbides, carbonitrides and nitrides. The general formula of the MAX phase is M_*n*+1_AX_*n*_, here M signifies an early transition metal (like Ti, Cr, V, Nb, *etc.*), A signifies an element from the groups 13–16 in the periodic table (like Al, As, Cd, P, *etc.*), and X signifies carbon and/or nitrogen.^88,89^ To differentiate this new group of 2D materials from graphene, the term MXene was coined, and the term MXene also applies for both the MXene fabricated from them and the original MAX phase.^90^ The MAX phase is hexagonally layered with *P*6_3_/*mmc* symmetry, where the X atom fills the octahedral sites, and the M_*n*+1_X_*n*_ layers are interleaved with A atom layers.^91^ The M–X link is ionic, metallic, and covalent in nature, whereas the M–A bond is predominantly metallic.^92^ Moreover, as the M–A bonds are weaker than M–X bonds, the loss of A element is easier by heating the MAX phase under vacuum^86,93^ and in molten salts,^94,95^ at high temperature. In recent days the incorporation of carbon materials like CNTs in the MXene layer has shown an enhanced result.^96,97^

### 4.1 0D carbon-MXene composite electrode materials for supercapacitors

Yu *et al.* demonstrated a novel strategy to use 2D Ti_3_C_2_T_*x*_ MXene as a conductive and flexible binder for one phase MXene-fused AC as supercapacitor's electrode material in organic electrolytes. Here they prepared a series of AC/MXene according to different mass proportions of AC to Ti_3_C_2_T_*x*_ and termed them AC/MXene-1:1, AC/MXene-2:1 and AC/MXene-4:1. In addition, they also prepared a conventional PVDF (polyvinylidene fluoride) bonded electrode (AC-PVDF) for electrochemical comparison. The specific capacitance of AM/MXene-1:1, AM/MXene-2:1 and AM/MXene-4:1 was found to be 88 F g^−1^, 126 F g^−1^, and 138 F g^−1^, respectively. The flexible composite (AC/MXene-2:1) shows a volumetric capacitance of 38 F cm^−3^. AC/MXene-2:1 shows a good capacitance retention of 65.4% and an improved conductivity of 29–166 S cm^−1^. At higher frequency, MXene films show the smallest ohmic resistance, thus indicating brilliant conductivity. The electrochemical properties of AC/MXene are highly improved because of its high surface area and abundant micropores.^98^ Habib and coworkers described the preparation of an onion-like carbon (OLC) coated titanium carbide MXene (Ti_2_C) electrode composite for a symmetric supercapacitor by doping Ti_2_CT_*x*_ with OLCs *via* sonicating them in *N*-methyl-2-pyrrolidone (NMD). In addition they have prepared 2 samples *i.e.* Ti_2_CT_*x*_//OLC (10% doping) and Ti_2_CT_*x*_//OLC (5% doping). 5% OLC-doped MXene exhibits 147 F g^−1^ specific capacitance, while 10% OLC-doped MXene exhibits only 92% F g^−1^ specific capacitance. Both the 5% and 10% OLC-doped MXenes maintain their original capacitance after 10 000 cycles, whereas the bare MXene loses 20% of its initial capacitance after 10 000 cycles. Moreover, the 5% OLC-doped MXene shows more than 100% capacitive retention. This is because of more activation, which might have occurred during high current cycling. Here the OLC not only increases the interlayer spacing to produce more ion transport channels but also increases the capacitance during cycles.^99^

### 4.2 1D carbon-MXene composite electrode materials for supercapacitors

A free-standing, flexible and conductive Ti_3_C_2_T_*x*_ MXene/carbon nanotube (CNT) composite electrode for acid supercapacitors has been assembled by Chen *et al. via* a simple vacuum filtration strategy. In addition they have prepared different MXene/CNT composites with different mass ratios of CNT:MXene-1 wt%, 5 wt% and 10 wt% and denoted them as MXene/CNT-1%, MXene/CNT-5% and MXene/CNT-10%. The GCD curve shows that the specific capacitance of MXene/CNT-5% is 300 F g^−1^, pure MXene is 290F g^−1^, MXene/CNT-1% is 295 F g^−1^, and MXene/CNT-10% is 285 F g^−1^. The low specific capacitance of MXene/CNT-10% is due to the reduction of active materials (MXene). Moreover, at 20 A g^−1^, MXene/CNT-5% delivers a performance retention of 92% over 10 000 cycles, confirming its excellent cycling performance. MXene/CNT-5% shows a slightly high interior resistance and increased slope at a lower frequency over 10 000 cycles at 20 A g^−1^ (from the EIS curve), which again confirms the rapid ion distribution properties of MXene/CNT-5% sheets and the outstanding conductivity of MXene. The high capacitance performance of the MXene/CNT composite is due to the intercalation of CNTs, which inhibits the restacking of MXene nanosheets.^100,101^ Levitt *et al.* incorporated Ti_3_C_2_T_*x*_ MXene with carbon nanofiber mats as an electrode material without binders or additives. Here they used a simple fabrication method of electrospun Ti_3_C_2_T_*x*_ MXene and polyacrylonitrile (PAN), and then the Ti_3_C_2_T_*x*_/PAN composite was carbonized to get a Ti_3_C_2_T_*x*_ MXene carbon nanofiber composite. Applying 1 M H_2_SO_4_, the electrochemical performances of the composite were analyzed in a three-electrode system, and Ag/AgCl is taken as the reference electrode . It is observed that the shape of the CV curves at 5 mV s^−1^ of high carbonization temperature and long-duration MXene carbon nanofiber composites (M_10_CNF800_1_, M_10_CNF600_2_, M_10_CNF600_6_, M_10_CNF700_6_) has more rectangular curves. At 10 mV s^−1^, the highest areal capacitance of 239 mF cm^−2^ was obtained for M_10_CNT800_1_, while the capacitance of bare CNF mats carbonized under the same conditions was found to be 75 mF cm^−2^ at 10 mV s^−1^. At highest frequency, Nyquist plots of Ti_3_C_2_T*_x_* MXene carbon nanofiber composite electrodes show a noteworthy difference as compared to pure-carbon fiber electrode. The larger semi-circle of pure carbon fibre represents greater charge transfer resistance than M_10_CNT800_1_.^79^ Li *et al.* prepared^17^ a porous MXene/carbon nanotube film through a simple process and showed a high specific capacitance of 401.4 F g^−1^ and sustained capacitance of 336.2 F g^−1^ at a high current density of 1000 A g^−1^, with excellent cycling stability of 99.0% after 20 000 cycles. The porous MXene/CNT film demonstrated outstanding electrochemical performance for supercapacitor applications due to its high specific capacitance, sustained capacitance, and cycling stability, effectively overcoming the limitations of strong interlayer van der Waals forces in MXenes.

### 4.3 2D carbon-MXene composite electrode materials for supercapacitors

**Fig. 5:**
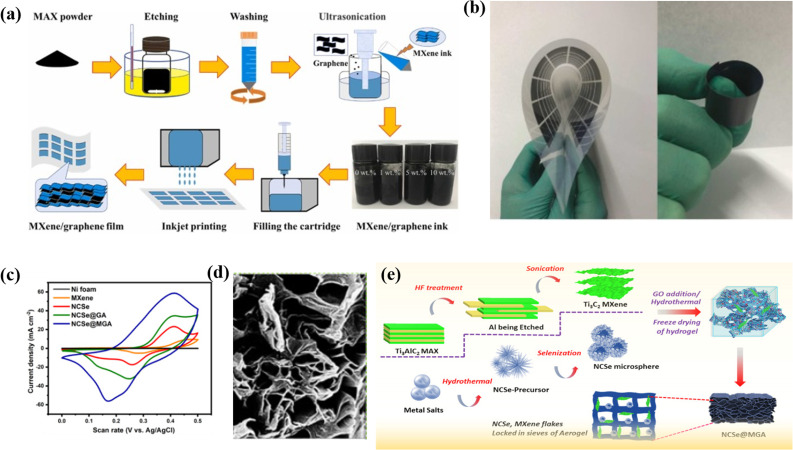
(a) A diagram showing the process of inkjet printing of MXene/graphene films, (b) the images of a flexible MXene/graphene composite electrode fabricated by inkjet printing process, (c) comparison of CV curves, (d) the interlinked network of NCSe@GA, and (e) a schematic representation of the production of Ti_3_C_2_ MXene, NCSe microspheres, and the creation of NCSe@MG.

Zhao and coworkers fabricated a two-dimensional titanium carbide and rGO (Ti_3_C_2_T_*x*_/rGO) composite by a LiF/HCL treatment. Herein they have prepared different Ti_3_C_2_T_*x*_/rGO fused compounds with varying ratios of Ti_3_C_2_T_*x*_/rGO-9, 7, 5, and 3 and termed them Ti_3_C_2_T_*x*_/rGO-9, Ti_3_C_2_T_*x*_/rGO-7, Ti_3_C_2_T_*x*_/rGO-5 and Ti_3_C_2_T_*x*_/rGO-3. The GCD curve reveals that with the increase in Ti_3_C_2_T_*x*_ in the composite (from 3 : 1 to 7 : 1), the specific capacitance of the composite also increases. However, the specific capacitance of Ti_3_C_2_T_*x*_ : rGO/9 : 1 decreases because the rGO is insufficient to stop all the Ti_3_C_2_T_*x*_ from agglomeration, which may cause insufficient usage of active materials and thus results in decreased capacitance performance. The Ti_3_C_2_T_*x*_/rGO-7 composite electrode shows the maximum *C*_sp_ of 154.3 F g^−1^ at 2 A g; in addition, a *C*_sp_ of 138 F g^−1^ is obtained at 6 A g^−1^. In addition, the manufactured composite has a better cycling performance, with a retention of 85% of its original capacitance over 6000 cycles. Here rGO nanosheets act as a conductive linker to link various 2D titanium carbide units.^102^ Fan *et al.* decorated binder-free and flexible, customized MXene@Holey graphene sheets by filtration of holey graphene oxide and alkalized MXene followed by annealing. During the preparation, alkali destroys the charge balance of MXene and HGO and replaces the –F group by the –OH group, which increases the Ti ratio and subsequently causes more pseudocapacitive reactions. The CV curve of MX-rHGO and pure MXene sheets at a 20 mV s^−1^ scan rate shows a broad peak. This broad peak confirms that the capacitance is due to the redox reaction of Ti atoms. Moreover, the as-prepared composite exhibits an outstanding rate capability as well as a notable volumetric capacitance of 1445 F cm^−3^. However, at 500 mV s^−1^, the MX-rHGO composite achieves a superior gravimetric capacitance of 302 F g^−1^ and retains an extraordinary rate capability of 69%. The electrochemical performance is due to the holey graphene, which prevents the aggregation of MXene and acts as a high nanopore conductivity network which eventually shortens the electrolyte ion transport pathway and facilitates the ion transmission.^103^ Flexible MXene/graphene composite electrodes were fabricated through an inkjet printing process by Wen *et al.*^104^ with printable graphene-modified MXene-based ink, showing excellent stability, a high volumetric capacitance of 183.5 F cm^−3^, and long cycle life, as well as a competitive energy density of 0.53 μW h cm^−2^ in a flexible supercapacitor. The flexible MXene/graphene composite electrodes demonstrated excellent performance as energy storage devices, offering prospects for the development of flexible and wearable energy storage devices with a combination of stability, high capacitance, long cycle life, and competitive energy density. A multifunctional Ti_3_C_2_ MXene@graphene composite aerogel with NiCo_2_Se_4_ was fabricated by Chaudhary *et al.*^16^ through a hydrothermal method, leading to an increase in the exposed electroactive surface area and fast multi-dimensional ion-phase transport. The composite showed a high specific capacity of 352.4 mA h g^−1^ and 99.6% initial coulombic efficiency, with a capacity retention of 91.5% after 5000 cycles. The material also demonstrated excellent electrocatalytic water splitting with low overpotentials and fast kinetics. The study presents a feasible strategy to design 3D electrode materials with optimal properties for various technological applications through the use of a multifunctional Ti_3_C_2_ MXene@Graphene composite aerogel. Fig. 5a displays a schematic diagram of inkjet printing of MXene/graphene films, comparative CV curves (Fig. 5b), an interconnected network of NCSe@GA (Fig. 5c), and a schematic illustration of the synthesis of Ti_3_C_2_ MXene, NCSe microspheres (Fig. 5d), and fabrication of NCSe@MG (Fig. 5e).

### 4.4 3D carbon-MXene composite electrode materials for supercapacitors

Guo *et al.* designed the fabrication of a Ti_3_C_2_T_*x*_-3D rGO framework binder-free aerogel architecture as an electrode material for supercapacitors *via* ascorbic acid-assisted hydrothermal treatments. Moreover, a sequence of Ti_3_C_2_T*_x_*/GO composites was prepared by adjusting the weight ratio of Ti_3_C_2_T_*x*_ and GO to 1, 2, 3 and 4, represented as Ti_3_C_2_T*_x_*@rGO-1, Ti_3_C_2_T*_x_*@rGO-2, Ti_3_C_2_T*_x_*@rGO-3, and Ti_3_C_2_T*_x_*@rGO-4, respectively. Ti_3_C_2_T_*x*_@rGO-4 has a larger area under the CV plot than the other electrode materials, which confirms its increased interlayer spacing and more active sites. In addition, at 1 A g^−1^ Ti_3_C_2_T_*x*_@rGO-4 displayed a *C*_sp_ of 313 F g^−1^. Moreover, at a high current density of 10 A g^−1^, it also exhibits a *C*_sp_ of 279 F g^−1^ and delivers an average retention of 78.8%. The Nyquist plot shows that the interfacial charge transfer resistance of Ti_3_C_2_T_*x*_@rGO-4 is less than that of the other electrode materials, which subsequently indicates its better ionic conductivity. Ti_3_C_2_T_*x*_@rGO-4 shows a retention capability of 89% after 6000 successive cycles. At a power density of 500 W kg^−1^ the as-prepared composite also showed an outstanding energy density of 7.5 W h kg^−1^. The exceptional capacitance properties of the composite can be ascribed due to the aerogel composite, which prevents the restacking of Ti_3_C_2_T_*x*_, increases the interlayer spacing, and facilitates the ion transport process.^105^ Wang *et al.* reported constructing a zinc ion hybrid supercapacitor (ZHSC) for the first time by fabricating porous Ti_3_C_2_T_*x*_ (3D MXene) decorated with reduced graphene oxide aerogel and zinc foil as the cathode and anode, respectively, in a 2 M ZnSO_4_ electrolyte. The rGO aerogel was generated using a hydrothermal process and then dipped in MXene before being freeze-dried to produce the MXene-rGO aerogel composite. At 0.4 A g^−1^ the ZHSC exhibited a maximum *C*_sp_ of 128.6 F g^−1^. In addition, at a high-power density of 279.9 W kg^−1^ the ZHSC showed a high electrochemical performance with a high energy density of 34.9 W h kg^−1^ and delivers a performance retention of 95% over 75 000 successive cycles at 5 A g^−1^ and a coulombic efficiency of 100%. These outstanding electrochemical properties are due to the special porous framework of the MXene-rGO aerogel composite, which prevents the stacking of MXene films and provides the aerogel with high electrical conductivity and outstanding electrical conductivity and hydrophilicity.^106^

MXenes have unique properties, such as electrical, optical, and mechanical properties, stability, and species that determine their electrochemical properties in supercapacitor applications. For example, Ti_3_C_2_T_*x*_ MXene thin films exhibit both transparency and conductivity, making them promising candidates for transparent conductive electrodes. Research has also begun to understand the energy-storage mechanisms that occur in electrochemical reactions and the factors determining the electrochemical behavior through the use of *in situ* techniques and modeling. The structure and surface chemistry of MXenes have a significant effect on their electrochemical performance, and efforts are being made to optimize them through the preparation conditions and post-treatment methods. MXenes are also being explored for use in various supercapacitor devices, such as 1D, 2D, and 3D symmetric or asymmetric supercapacitors, microsupercapacitors, and transparent supercapacitors. However, challenges remain in terms of improving the electrochemical performance of MXenes and promoting their practical applications. They include the need for safe and low-cost synthesis methods and a deeper understanding of effective anions and cations during the etching process.

## 5. Layered double hydroxides (LDHs)

**Fig. 6:**
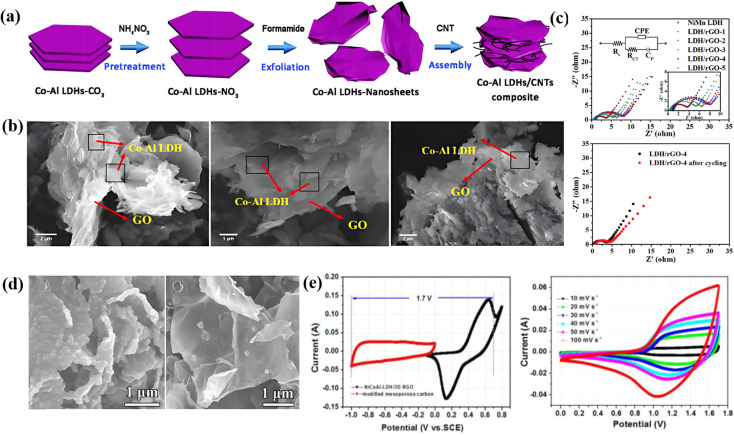
(a) The schematics of each process of assembling the exfoliated Co–Al LDHs-CNT composite. (b) The SEM images with different mass ratios. (c) Corresponding Nyquist plots and the equivalent circuit before and after cycling. (d) FESEM images of ZIF-67/GO, and (e) CV and GCD curves of LDH/rGO-4.

Layered double hydroxides are a class of inorganic materials constituted by two dimensional, extremely tunable brucite-like layered structures. These inorganic substances have levels of metal hydroxides, which are positively charged, and the presence of anions plays an important role in neutrality. The general formula for LDHs is [M_1−*x*_^2+^M_*x*_^3+^(OH)_2_]^*x*+^(A^*n*−^)*x*/*n*·*m*H_2_O, where M^2+^ denotes divalent cations such as Mg^2+^, Ni^2+^, Co^2+^, Zn^2+^, *etc.*, M^3+^ denotes trivalent cations such as Al^3+^, Fe^3+^, *etc.*, An represents an anion and can be SO_2_^−4^, CO_2_^−3^, F^−^, Cl^−^, OH^−^ or NO^−3^ and the value of *x* varies from 0.2 to 0.33.^107^ LDHs are also known as hydrotalcite-like compounds (Mg_6_Al_2_(OH)_16_CO_3_·4H_2_O), obtained from stacking brucite-like layered crystal structures. The positive charge of this hydrotalcite-like compound is due to partial substitution of Mg^2+^ by Al^3+^; however, this positive charge is compensated for by the anions situated at the interlamellar spaces^108^ [as shown in Fig. 6a]. Furthermore, the An present in the interlamellar space is exchangeable. Nowadays, LDHs are attracting tremendous interest in different potential applications because of their facile synthesis, chemical versatility, high redox activity, low cost, and thermal stability.^109^ However, their composites with graphene, carbon nanotubes and CNFs have attracted tremendous research interest for high-performance supercapacitors because of their exceptional merits due to a combination of special properties of their parental materials. Fig. 6 displays a comprehensive illustration of the exfoliated Co–Al LDHs-CNT composite assembly process, including the schematics of the process (Fig. 6a), SEM images with varying mass ratios (Fig. 6b), Nyquist plots and equivalent circuits before and after cycling (Fig. 6c), FESEM images of ZIF-67/GO (Fig. 6d), and CV/GCD curves of LDH/rGO-4 (Fig. 6e).

### 5.1 0D carbon-LDH composite electrode materials for supercapacitors

Wei and co-workers employed a one-step solvothermal procedure to make unique ultra-thin nanoplate like NiAl-LDHs/carbon quantum dot (CQDs/NiAl-LDHs) architecture nanosheets as a supercapacitor electrode material. At 2 A g^−1^ the as-prepared architecture produced a superior capacitance of 1794 F g^−1^. In addition, at 20 A g^−1^ a remarkable rate capability of 967 F g^−1^ has been reported for the architecture. It also shows its noteworthy electrochemical performance, and a remarkable retention of 93% was also observed over 1500 cycles. Fig. 6b presents an EIS plot of the CQDs/NiAl-LDH architecture and pure NiAL-LDH electrode, which shows that the contact resistance of the CQDs/NiAl-LDH architecture is smaller (0.84 ohms) than that of bare NiAl-LDH (1.26 ohm). The notable electrochemical characteristics of the as-prepared architecture can be ascribed to the synergetic combination of CQDs and NiAl-LDH.^110^ Qui and coworkers prepared a flower ball-like composite of histidine-functionalized graphene quantum dots/Ni–Co LDH (His-GQD/LDH) by using a microwave technique. They prepared His-GQD by using histidine and citric acid as carbon sources *via* pyrolysis. An exceptional *C*_sp_ of 1526 F g^−1^ was shown by the His-GQD/LDH electrode material. Furthermore, a good cycling retention of 82.36% was measured over 2000 cycles at 10 A g^−1^, indicating its excellent cycling performance. Moreover, they constructed an unsymmetric supercapacitor system by applying activated carbon (AC) and His-GQD/LDH as the negative electrode and positive electrode, respectively, which displays a cycling performance of 91.13% retention after 6000 cycles and maintains an energy density of 48.89 W h kg^−1^ at a power density of 0.80 kW kg^−1^. The synergistic effect of LDH and His-GQD, which considerably boosts the material's conductivity and specific surface area, can be attributed to the His-GQD/LDH composite material's outstanding electrochemical characteristics.^111^

### 5.2 1D carbon-LDH composite electrode materials for supercapacitors

Luo *et al.* described the construction of a series of NiAl-LDH and CNT composite electrode materials by the combination of nickel aluminum layered double hydroxide (NiAl-LDH) and CNTs. The NiAl-LDH/CNT composite was termed LDH-*X*, where *X* denotes the mass of CNTs as 10, 20, 30, and 40 mg. CV curves of various electrodes (LDHs, LDH-10, LDH-20, LDH-30, and LDH-40) were scanned at 5 mV s^−1^. LDH-30 shows a larger integral area in the CV plot, confirming that a suitable amount of CNTs can produce more active sites and can enhance the capacitive properties of the engineered material. In addition, the GCD curve at 2 A g^−1^ indicates that LDH-30 produced the best capacitance value of 2447 F g^−1^. Moreover, it exhibited 90.1% retention after 2500 cycles, confirming its extraordinary cycling stability. These good electrochemical properties of the NiAl-LDH/CNT composite can be ascribed to the synergetic effect of NiAl-LDHs and CNTs.^112^ Yu *et al.* developed a sandwich-like structured Co–Al LDHs/carbon nanotube (Co–Al LDH/CNT) composite *via* an electrostatic assembly process. A high specific capacitance of 884 F g^−1^ was observed for the as-built composite at 5 mA cm^−2^. The CNTs exhibited a specific capacitance of 96 F g^−1^, and Co–Al LDHs showed a capacitance performance of 547 F g^−1^ at a 5 mA cm^−2^ charging–discharging current. Furthermore, at 10 mA cm^−2^, the Co–Al LDH/CNT composite displayed better cycling stability of 88% over 2000 cycles. Electrochemical impedance spectroscopy (EIS) shows that Co–Al LDH/CNT possesses a semicircle with a smaller diameter at the high frequency region than the bare Co–Al LDHs, indicating a smaller charge transfer resistance (*R*_ct_) of Co–Al LDH/CNT than Co–Al LDHs. Additionally, an asymmetric supercapacitor was built by employing Co–Al LDH/CNT and AC as the positive electrode and negative electrode, respectively. The asymmetric supercapacitor produced a specific capacitance of 80.6 F g^−1^ at 5 mA cm^−2^ and retention of 88.9% after 1000 cycles, and at a power density of 444.1 W kg^−1^, it showed an energy density of 28 W h kg^−1^.^113^

### 5.3 2D carbon-LDH composite electrode materials for supercapacitors

Li *et al.* developed an environmentally friendly, highly effective, and facile solid-state exfoliation method to exfoliate CoAl LDH and graphene oxide and prepared a series of CoAl LDH/rGO by adjusting the mass ratio of Co-Al LDH/GO = 1 : 1 (Co-Al LDH/GO-1), Co-Al LDH/GO = 3 : 1 (Co-Al LDH/GO-3), and Co-Al LDH/GO = 5 : 1 (Co-Al LDH/GO-5) through reduction treatment. The CV curves of the various composites at 10 mV s^−1^ show that CoAl LDH/rGO-3 has the highest *C*_sp_ value of 1560.0 F g^−1^. For clarity, they used the GCD method at 0–0.5 V. The *C*_sp_ values for CoAl LDH/rGO-3, CoAl LDH/rGO-1, CoAl LDH/rGO-5, CoAl LDH, and rGO at 1 A g^−1^ were 1492.0, 1062.2, 1389.4, 523.8, and 40.2 F g^−1^ respectively. Additionally, they observed that the capacitance performance of the various as-prepared architectures decreased with an increase in current density ranging from 1 to 20 A g^−1^ because the liquid ions and active surface of the electrode may not provide an efficient contact. Furthermore, the CoAl LDH/rGO-3 composite exhibited outstanding electrochemical performance, retaining 94.3% of its capacitance after 5000 cycles and maintaining 799.6 W kg^−1^ power density and 44.6 W h kg^−1^ energy density. Here the good electrochemical properties of CoAl LDH/rGO-3 can be ascribed to the synergism between rGO and CoAl LDH, where the CoAL LDH is prevented from restacking and agglomeration which results in good capacitance performance.^114^ Huang *et al.* used a simple solvothermal approach to create a three-dimensional flower-like hierarchical structure of the NiMn LDH/rGO composite with varied mass loadings (GO-1, 3, 5, 7, and 9 mg). The CV curves of the different composites show that LDH/rGO-4 exhibited better electrochemical properties than the other samples because it had a larger surface area. Meanwhile the GCD curve again confirms its improved capacitance properties owing to its longer discharge time. In addition, the as-prepared LDH/rGO-4 electrode material exhibited superior capacitance values of 1500, 1216, 1086, 920, 812, and 680 F g^−1^ at current densities of 1, 2, 3, 5, 7, and 10 A g^−1^, respectively. The *R*_ct_ of LDH/rGO-4 was determined to be 3.42 ohm, which is much lower than that of the other samples obtained from the Nyquist plot, revealing its fast charge transfer during the reaction process. Moreover, it displayed an outstanding retention of 90.5% after 5000 cycles at 20 A g^−1^. They also reported an unsymmetrical supercapacitor device by employing rGO and LDH/rGO-4 as negative and positive electrodes, which presented a *C*_sp_ of 82.5 F g^−1^ at a current density of 0.5 A g^−1^ and an outstanding energy density of 29.3 W h kg^−1^. The electrochemical behavior of LDH/rGO-4 can be attributed to its unique framework, abundant substrate area, and favorable pore size.^115^

**Fig. 7:**
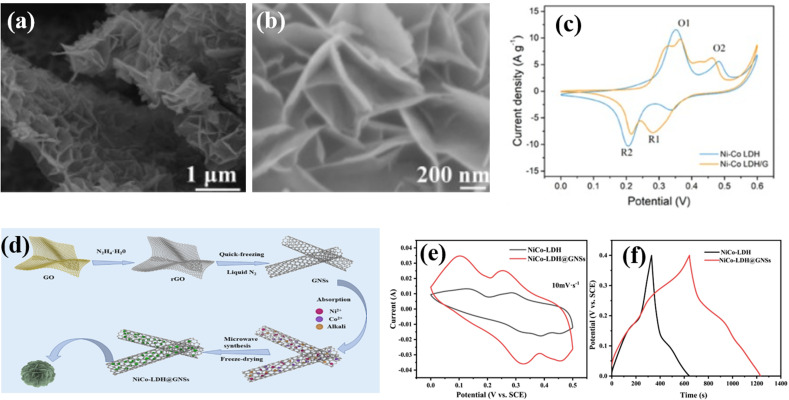
(a) Low and (b) high magnification SEM images of the Ni–Co LDH/G composite, (c) a comparison of CV curves, (d) a schematic of the synthesis process of NiCo-LDH@GNSs, (e) CV curves of both pristine NiCo-LDH and NiCo-LDH@GNSs, and (f) CV curves of activated carbon and the NiCo-LDH@GNS composite at a scan rate of 10 mV s^−1^.

Zhang *et al.* designed nickel-cobalt LDH nanofilms coated with a reduced graphene oxide support (NiCo-LDH/rGO) as electrode materials *via* solvothermal treatment. At 1 A g^−1^, the as-synthesized nanostructure delivered a capacitance of 1675 F g^−1^ and an 83.8% retention at 10 A g^−1^. However, at 1 A g^−1^, NiCoLDH exhibited a capacitance of 920 F g^−1^ and only 81.5% retention at 10 A g^−1^. Moreover, they prepared an asymmetric supercapacitor system using activated carbon (AC) and NiCo-LDH/rGO as the electrode material, displaying a power density of 3747.9 W kg^−1^ and a remarkable energy density of 49.9 W h kg^−1^. The excellent electrochemical performance of the generated nanocomposite can be due to the collective interaction of conductive graphene and electro-active LDH, in which graphene promotes conductivity while the supporting NiCo-LDH effectively prevents the self-aggregation of graphene.^116^ A high-efficiency microwave liquid synthesis method was used to prepare a honeycomb-structured Ni–Co LDH nanosheets on graphene (Ni–Co LDH/G) composite by Zou *et al.*, which improved the rate capability and conductivity of the Ni–Co LDH material. The Ni–Co LDH/G composite shows promising results as a supercapacitor electrode with a high energy density (40.6 W h kg^−1^) and stable cycling lifespan (83.2% capacity retention after 10 200 cycles). Kuang *et al.* reported the design and synthesis of an array core–shell heterostructure graphene nanoscroll composite with NiCo-LDH nanoflakes for improved electrochemical performance in energy storage devices.^117^ The NiCo-LDH@GNS electrode shows a high specific capacitance of 1470 F g^−1^ at 1 A g^−1^ and a long cycle life with 81.6% retention after 1000 cycles due to its advantageous nanostructure. Zou *et al.*^118^ used the microwave liquid synthesis method to prepare honeycomb-structured Ni–Co LDH nanosheets on graphene, which improved the conductivity and rate capability of the material, leading to a high-energy density hybrid supercapacitor. The use of graphene in the synthesis of Ni–Co LDH/G composites significantly improved their conductivity, resulting in a high-rate capability and a high energy density of 40.6 W h kg^−1^, making it a promising electrode material for commercial applications. Fig. 7a and b include the SEM images of the Ni–Co LDH/G composite, a comparison of CV curves (Fig. 7c), a schematic of the synthesis process of NiCo-LDH@GNSs (Fig. 7d), CV curves of pristine NiCo-LDH and NiCo-LDH@GNSs (Fig. 7e), and CV curves of AC and the NiCo- LDH@GNS composite at a scan rate of 10 mV s^−1^ (Fig. 7f).

### 5.4 3D carbon-LDHs composite electrode materials for supercapacitors

A novel Ni/Mn layered double hydroxide-encapsulated graphene foam nanoarchitecture was constructed by Li *et al.* using a facile and simple low-temperature chemical coprecipitation method. They prepared Ni/Mn LDH/GF nanocomposites with different Ni : Mn ratios, and Ni/Mn LDH/GF with Ni : Mn = 6 : 4 exhibited superior specific capacitances of 2380, 2314, 2081, 827, 1567, 1415, and 1315 F g^−1^ at current densities of 1, 2, 3, 5, 10, 15, and 20 A g^−1^, respectively. In addition, at a high charging–discharging current of 20 A g^−1^, a high capacitance performance of 1315 F g^−1^ was produced, which reveals its good rate capability. Additionally, they engineered an entirely solid-state flexible unsymmetrical ultracapacitor by incorporating Ni/Mn LDH@graphene foam as the positive electrode and activated carbon fabric as a negative electrode which exhibited a high power density of 1.10 kW kg^−1^ and high energy density of 138.83 W h kg^−1^ and maintained cycling stability of 94.9% over 1000 cycles. The larger substrate area and higher interfacial strength of the engineered composite contribute to its electrochemical performance.^119^ Zhang *et al.* developed a facile hydrothermal treatment to prepare a three-dimensional (3D) hybrid aerogel made of CoAl LDH nanosheets and 3D graphene aerogels. The capacitive properties of the CoAl LDHs/graphene hybrid aerogels (Co–Al LDHs/GHAs) as-synthesized were exceptional, with a specific capacitance of 640 F g^−1^ at a low current density of 1 A g^−1^. Furthermore, at high current densities of 10 and 20 A g^−1^, a strong *C*_sp_ of 530 F g^−1^ and 305 F g^−1^, respectively, was maintained. Furthermore, the three-dimensional hybrid aerogel composite showed 97% superior cycling performance after 10 000 cycles. The synergetic effect of Co–Al LDHs, which give a short diffusion pathway, and 3D graphene aerogel, which provides superior electrical conductivity, is responsible for the increased electrochemical performance.^120^ Bai *et al.* reported the manufacture of NiCoAl LDH/three-dimensional rGO nanopetals using a deep dip-coating approach collectively by alkaline reduction and hydrothermal reactions, which resulted in increased specific capacitance, better cycling stability, outstanding rate capability, and electrochemical capacitance. Furthermore, using sucrose as the carbon source, modified mesoporous carbon (MMC) was successfully synthesized *via* a simple template sacrificial approach and then modified using HNO_3_. MMC yielded the highest *C*_sp_ of 289 F g^−1^ when employed as the negative electrode in an ultracapacitor system. Furthermore, an entirely solid-state asymmetric ultracapacitor with a negative electrode of modified mesoporous carbon and a positive electrode of NiCoAl-LDH/3D rGO was fabricated. The capacitive behavior of the as-prepared unsymmetric system is excellent, which produced an excellent power density of 8650 W kg^−1^ along with 93.6% retention over 4500 cycles and a fantastic energy density of 57.1 W h kg^−1^.^121^

**Fig. 8:**
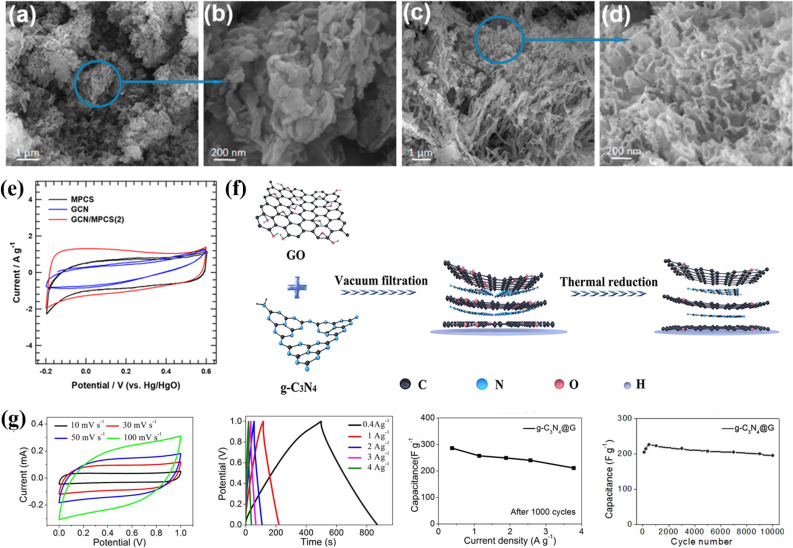
(a–d) SEM images of BCN and TCN-200. (e) CV curves of pristine GCN, MPCS, and GCN/MPCS. (f) Schematic diagram of the synthesis of the graphene/g-C_3_N_4_ composite, and (g) CV, GCD curves and corresponding capacitance value.

LDHs are a class of materials studied for their pseudocapacitance properties in supercapacitor applications. The factors affecting the pseudocapacitance performance of LDHs can be divided into three main aspects: the interaction between OH^−^ and LDHs, the exposed ratio of active atoms, and the basic electrical conductivity of LDHs. Current research focuses on modifying these three aspects through various strategies such as composition regulation, modification of active sites, enhancement of electrical conductivity, and other methods such as modifying the morphology and structure. However, there are still many aspects that require improvement for the modification and pseudocapacitance study of LDHs. They include further research on the effect of the pseudocapacitance process on the structure and components of LDHs, more research on doping and vacancies in LDHs, and innovation in preparation methods to understand the combinability of multiple modification methods. Additionally, research is needed to understand how to make the best use of the structural merits of LDHs, such as their hollow nano-architecture, large specific surface area, and tunable interlayer spacing. Fig. 8a–d show the SEM images of boron-doped carbon nitride and TCN-200 (tungsten-doped carbon nitride), which show the morphological features of the materials. The CV curves in Fig. 8e show electrochemical behaviour of pristine GCN (graphitic carbon nitride), MPCS (mesoporous carbon sphere), and GCN/MPCS (graphitic carbon nitride/mesoporous carbon sphere). A schematic diagram (Fig. 8f) of the synthesis process of the graphene/g-C_3_N_4_ (graphene-coated triazine-based graphitic carbon nitride) composite is also included in the figure. This diagram provides an understanding of the composite formation. Fig. 8g shows the CV and GCD (galvanostatic charge/discharge) curves as well as the corresponding capacitance value of the graphene/g-C_3_N_4_ composite, to evaluate its electrochemical capacitance performance. Fig. 8 provides a comprehensive overview of the morphological and electrochemical characteristics of these materials.

## 6. Graphitic carbon nitrides (g-C_3_N_4_)

Carbon nitrides are metal-free polymetric materials, generally consisting of nitrogen and carbon. They can be obtained by the substitution of carbon atoms by nitrogen atoms from carbon materials.^122^ Carbon nitride has a long history, which can be dated back to 1834 when Berzelius synthesized a material and named “melon” (linear polymer of linked tri-*s*-triazine) which was later reported by Liebig.^123^ Five possible phases of carbon nitride (C_3_N_4_) were reported by Teter *et al.* in the year 1996, namely alpha-C_3_N_4_, g-C_3_N_4_, pseudocubic-C_3_N_4_, cubic-C_3_N_4_, and beta-C_3_N_4_. Out of the several phases, g-C_3_N_4_ has received the most attention because of its plentiful nitrogen (N) and carbon (C), low cost, easy synthesis, and environmentally sound features. g-C_3_N_4_, an n-type semiconductor, has a graphite-like structure. Each planar sheet of g-C_3_N_4_ has a two-dimensional stacked structure with sp^2^ hybridized conjugated nitrogen and carbon atoms and a pie conjugated electronic structure. In g-C_3_N_4_, the stacking distance is 0.319 nm, much lower than that of graphite (0.335 nm). This increased packing density binds the layers of g-C_3_N_4_ more strongly. Furthermore, condensed tri-*s*-triazine (C_6_N_7_) has been discovered to be the fundamental subunit for the production of g-C_3_N_4_.^124^ Graphitic carbon nitride is gaining popularity in various fields, like sensing, energy storage and catalysis because of its non-toxicity, excellent physiochemical properties, absence of metals, lamellar structure, and chemical inertness.^125^ Carbon materials like graphene, AC, and reduced graphene oxide have some drawbacks such as intrinsic electrostatics, surface charging mechanism and severe agglomeration, which hinders the energy density and specific capacitance; thus, fabricating a C/g-C_3_N_4_ composite as an electrode material has been implemented.^126–129^

### 6.1 0D carbon/g-C_3_N_4_ composite electrode materials for supercapacitors

Lin *et al.* assembled carbon-plated g-C_3_N_4_ nanotubes and labeled them C-TCN by the simultaneous thermal polymerization of urea followed by carbonization of glucose. A composite of TCN-200 with a high nanotube content was also prepared by setting the mass balance of glucose/urea to 1/30. In addition, a series of C-TCN were prepared with different heating temperatures and termed TCN-200-T*X* (*X* = 300, 400 & 500), and a series of TCN-Y were also prepared according to different glucose contents, where *Y* = 0, 50, 100, 300, 500, 1000 or 2000. At 50 mV s^−1^, the CV curves of TCN-*Y* and bulk carbon nitride (BCN) indicate that TCN-200 exhibits the highest capacity because it possesses the largest CV curve. This large capacity can be attributed to the nanotubular framework, which enhanced the specific surface area, and the conductivity of the C-TCN coating was improved. At 1 A g^−1^, the GCD curves of TCN-*Y* and bulk carbon nitride show a certain curvature, confirming its pseudocapacitive characteristics caused by the large quantity of nitrogen present in g-C_3_N_4_. Moreover, TCN-200 also had a *C*_sp_ of 241.6 F g^−1^ at 1 A g^−1^, which was 1.9 times greater than that of BCN (*i.e.*,124.4 F g^−1^). It also maintained 85.7 percent of capacitance over 1000 cycles at 2 A g^−1^. This decrease in capacitance performance is due to some damage to the nano-tubular structure during the charge–discharge reaction.^130^ Oh and coworkers reported the preparation of a g-C_3_N_4_ loaded mesoporous carbon sphere nano-composite (GCN/MPCS) *via* facile hydrothermal treatment. Here the MPCS was obtained from carboxymethyl cellulose. In addition, a series of GCN/MPC nano-architectures were prepared by regulating the mass balance of MPCS and GCN to 3 : 1, 2 : 1, and 1 : 1 and termed GCN/MPCS(3), GCN/MPCS(2), and GCN/MPCS(1) respectively. CV profile of GCN/MPCS (2), MPCS, and GCN are obtained in a 6 M KOH electrolyte at a scan rate of 5 mV s^−1^ over a potential range of −0.2 to 0.6 V (*vs.* Hg/HgO). It has been detected that the GCN & MPCS electrode exhibits a rectangular CV curve; in addition, a quasi-rectangular reversible electron transfer progression is also noticeable, which confirms the double-layer type capacitance mechanism. Furthermore, the cyclic voltammogram profile of GCN/MPCS(2) shows enhanced double-layer capacitance characteristics. A *C*_sp_ of 131.65 F g^−1^, 231.39 F g^−1^, and 352.44 F g^−1^ was reported for GCN, MPCS, and GCN/MPCS(2) respectively at 5 mV s^−1^. Furthermore, it was observed that with the rise in the scanning speed of 5 mV s^−1^ to 500 mV s^−1^, a high rate performance of 80.05% is also observed for GCN/MPCS(2). In addition, only 11.56% loss in capacitance was observed over 3000 cycles at 1 A g^−1^. The high electrochemical performance of the GCN/MPCS nanocomposite can be ascribed to the porous structure effect of MPCS.^131^ The results from Density functional theory (DFT) calculations and capacitance contribution fitting by Qiu showed that adding g-C_3_N_4_(CN) to LDH significantly increases the specific capacitance of the electrode. The supercapacitor assembled using the CN-LDH electrode displayed a maximum energy density of 50.63 W h kg^−1^ at a power density of 0.80 kW kg^−1^, and successfully lit several LEDs, making it a potential material for supercapacitor applications.

### 6.2 1D carbon/g-C_3_N_4_ composite electrode materials for supercapacitors

Chao *et al.* constructed a carbon nanotube/graphitic carbon nitride electrode material for an all-solid-state supercapacitor *via* ultrasonication. The as-prepared composite exhibited enhanced electrical conductivity, a high nitrogen content, and a large specific surface area. At 1 A g^−1^, the as-fabricated all-solid-state supercapacitor exhibited an outstanding electrochemical performance of 148 F g^−1^, high energy capacity, and 93% cycling performance over 10 000 cycles at 1 A g^−1^. Moreover, at a power density of 397.98 A, an energy density of 13.16 W h kg^−1^ was observed. CV curves of carbon nanotube/graphitic carbon nitride electrode material show rectangular shapes with slight disorder under different scan rates, which confirms its pseudocapacitive behavior. Here, nitrogen induces pseudocapacitive behavior, which eventually enhances capacitive properties. The high capacitive properties are due to the large surface area with porosity, which acts as an ion accommodation reservoir during the charge–discharge mechanism.^132^ NiCo-layered double hydroxide (NiCo-LDH) self-growing nanosheet arrays decorated with graphitic carbon nitride (g-C_3_N_4_) were found to be a high-performing potential electrode material for supercapacitors, with a specific capacitance of 1936.36 F g^−1^ at 1 A g^−1^. A convenient avenue for the fabrication of CuFe-ZIF@ZIF67-derived carbon framework (CuFe/N–C@Co/N-CNTs) incorporating graphitic carbon nitride (CuFe-ZIF@ZIF67@gCN) and graphene oxide (CuFe-ZIF@ZIF67@GO) as bifunctional catalysts was presented by Kamali *et al.* CuFe/N–C@Co/N-CNTs@GO showed superior performance as an efficient bifunctional electrocatalyst due to the synergistic effects of CuFe/N–C@Co/N-CNTs and graphene oxide, and revealed remarkable energy storage capability, abundant porosity, high conductivity, outstanding electron and mass transfer, large surface area, and excellent stability with 94.4% current retention after 20 000 s. The above result shows that CuFe/N–C@Co/N-CNTs@GO nannocomposite displays outstanding electrochemical performance in supercapacitors as well as oxygen reduction reactions.

### 6.3 2D carbon/g-C_3_N_4_ composite electrode materials for supercapacitors

A flexible, all-solid-state supercapacitor system was constructed by Qu *et al.* by using a graphene/g-C_3_N_4_ (GNP) composite as the electrode material *via* vacuum filtration followed by the thermal reduction method. They prepared a series of GNP composites under the various temperatures of 250, 350 and 450 °C and termed them GNP-250, GNP-350 and GNP-450, respectively. To study the capacitive behavior of the as-prepared nanomaterial, CV and GCD measurements have been implemented. A high mass-specific capacitance and high specific areal capacitance of 325 F g^−1^ and 1500 mF cm^−2^ respectively were recorded. The CV curves of GP-0, GNP-250, GNP-350 and GNP-450 in the −1 to 0 V potential window indicate that the area covered by GNP-350 is larger than that of any other electrode material, indicating its larger capacitance which is about three times greater than that of pure graphene paper. Moreover, the GCD curves of the same samples at a current density of 1 mA cm^−2^ show that GNP-350 has the largest persistent charge–discharge time. In addition, an all-solid-state symmetric sandwich supercapacitor (ASSSCs) was constructed by employing GNP-350 as the electrode in which PVA-KOH gel is employed as the electrolyte, resulting in an areal capacitance of 425 mF cm^−2^ and maintains 95% of initial capacitance over 5000 cycles. Furthermore, a maximum energy density of 0.075 mW h cm^−2^ has been reported. Here g-C_3_N_4_ has been used as a spacer for graphene to avoid assembling between graphene layers, which finally improves the specific substrate area of the electrolyte and improves the capacitance performances.^133^ Chen *et al.* created a symmetrical supercapacitor device using a one-step hydrothermal process to turn graphene and binder-free g-C_3_N_4_ into a 3D linked network. The as-prepared composite (g-C_3_N_4_@G) produced a specific capacitance of 264 F g^−1^. In addition, a high-power density and energy density of 4.0 kW kg^−1^ and 30 W h kg^−1^, respectively, were reported. Moreover, even at 4.0 A g^−1^, the composite managed to maintain a *C*_sp_ of 211 F g^−1^. The capacitance plot of the SC device shows that the g-C_3_N_4_@G device shows an excellent longer phase electrochemical stability after 10 000 cycles. For further study of electrochemical performance, the EIS plots of a g-C_3_N_4_@G and 3DG based supercapacitor device were obtained, which clearly indicate that the g-C_3_N_4_@G based supercapacitor has a little lower *R*_s_ value as compared to 3DG at high frequencies. The 3DG based supercapacitor device exhibits a capacitance of 152 F g^−1^. Herein, the high electrochemical performance of g-C_3_N_4_@G is due to the 3D interconnected network structure of g-C_3_N_4_@G, which provides a hierarchical structure, induces multi-way electron transfer, and facilitates charge transfer.^58^ Recently Antil *et al.* developed a unique g-C_3_N_4_. 7/N-doped graphene nanocomposite electrode material which shows robust charge storage characteristics *via* reducing the interfacial resistivity. The device delivered a high areal capacitance of 141 mF cm^−2^, power density of 0.70 mW cm^−2^ and an energy density of 0.047 mW h cm^−2^, with 98.9% capacity retention at 2.2 V potential till 18 000 cycles and nearly 95% coulombic efficiency till 20 000 cycles. Additionally, the device showed the stability of capacity at any bending angle.^134^

The performance of supercapacitors mainly depends on the nature of the electrode materials, electrolyte, and voltage window of the material used. There is a growing research focus on developing new electrode materials to enhance supercapacitive performance. One such material is graphitic carbon nitride (g-C_3_N_4_), which is a unique analogue of graphene with nitrogen as a heteroatom. Despite its limitations, such as low surface area and conductivity, g-C_3_N_4_ has shown excellent mechanical stability and flexibility. Studies are underway to alleviate these shortcomings by modifying the structural network of g-C_3_N_4_ or by coupling it with other nanocomposites. The combination of g-C_3_N_4_ with other materials, such as transition metal oxides, conducting polymers, and carbon materials, has led to an enhancement in the surface area and redox reaction at the electrode–electrolyte interface. These g-C_3_N_4_ composites exhibit excellent supercapacitive performance, but further research is needed to optimize their use in practical applications and explore new combinations with metal phosphides and other functional groups.

## 7. Metal–organic frameworks (MOFs)

**Fig. 9:**
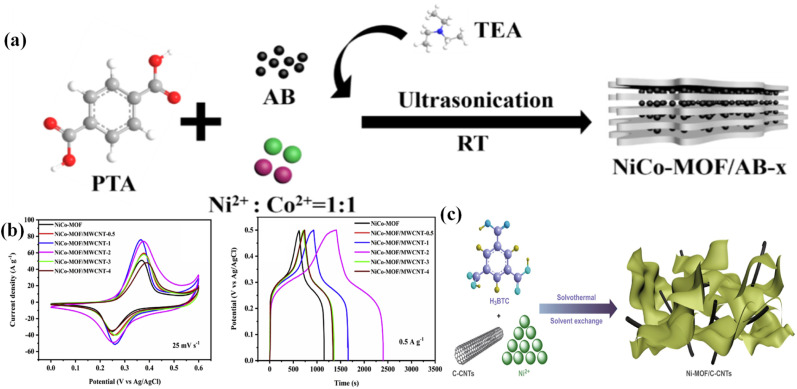
(a) Illustration of the synthesis process of NiCo-MOF/AB composites in a schematic form. (b) CV and GCD curves of NiCo-MOF and NiCo-MOF/MWCNT electrodes were recorded in a three-electrode system, and (c) schematic illustration was provided for the synthesis of ultrathin Ni-MOF/C-CNT nanosheets.

MOFs (also known as coordination polymers) are a class of novel developed porous materials composed of metal centers and electron-donating organic ligands,^135^ and have drawn wide interest in numerous fields such as catalysis, drug delivery, adsorption, energy storage, and sensors.^136–139^ MOFs are now being increasingly employed as supercapacitor electrode materials owing to their abundant specific surface area, tunable permeability, and outstanding chemical and physical properties.^140^ In addition, MOFs have emerged as the latest class of materials for energy storage applications because of their combined redox-active metal centers and high surface area.^141^ To date, Co-, Ni-, Mn-, Zn-, Fe-, Cd-, In-, Cu-, and Zr- based MOFs have been reported for supercapacitor applications (Fig. 9).^142^

Moreover, a tremendous effort has been made to research MOFs and MOF-based derivatives in supercapacitor applications, where M-PTA (PTA: *p*-benzenedicarboxylate, also known as terephthalate), M-BTC (BTC: 1,3,5-benzenetricarboxylate) and ZIFs (zeolitic imidazolate framework) are most widely investigated.^143^ MOFs are constructed by linking organic and inorganic building units by coordination bonds, in which the inorganic units are mainly metal ions or clusters and widely denoted as SBUs (Secondary Building Blocks), whereas organic units (linkers or bridging ligands) are mainly carboxylates or other organic anions like sulphonate, phosphate, and heterocyclic compounds. The framework topology of MOFs can be determined by the combination of the orientation of organic linkers and SBUs (connectors). For example, a 4-connected tetrahedral cluster and ditopic linear linker form a diamondoid net. And 6-connected octahedral clusters along with a ditopic linear linker form a cubic net.^144^ Based on structural functionalities MOFs are categorized into several types such as two-dimensional (2D) MOFs, porous coordination networks, polyoxometalate MOFs (POMOFs), entangled MOFs, mew emerging MOFs, and heterometallic MOFs. 2D MOFs are further classified as layered MOFs, graphene analogue conductive MOFs, and MOF-based nanosheets according to their performance.^145^ Although MOFs exhibit good capacitive properties, their poor cycling stability and electrical conductivity hinder their practical application.^141,146^ Therefore morphological/structural engineering of MOFs and fabricating with stable and conductive materials have attracted increasing interest in the research field to solve such challenges. The synthesis process of NiCo-MOF/AB composites was illustrated in a schematic form (figure a). The CV and GCD curves of NiCo-MOF and NiCo-MOF/MWCNT electrodes were recorded in a three-electrode system (figure b). The synthesis of ultrathin Ni-MOF/C-CNT nanosheets was also in a schematic form (figure c). The basic framework of conductive 2D MOFs, in which layers are mounted towards the *c* direction to produce naturally porous crystallites with pores of around 2 nm in diameter, is depicted in this diagram. Metal atoms and organic ligands comprise the honeycomb lattice of the 2D layers. Ni_3_(HITP)_2_ and Cu_3_(HHTP)_2_ have M/X = Ni/NH and Cu/O, respectively.

### 7.1 0D carbon/2D MOF composite electrode materials for supercapacitors

Liu *et al.* used a simple ultrasonic technique to prepare a NiCo-MOF/acetylene black nanostructure at ambient temperature. NiCo-MOF/AB-*x* is the name given to the as-prepared sample, where *x* represents the (acetylene black) mass. The acetylene black masses were taken as 10, 5, 2.5, and 0 mg, and the representative composites were called NiCo-MOF10, NiCo-MOF/AB5, NiCo-MOF/AB2.5, and NiCo-MOF, respectively. At 1 A g^−1^, the NiCo-MOF/AB-5 composites produced a supreme *C*_sp_ of 916.1 F g^−1^ with 94.1% initial capacitance retention over 3000 cycles. At 1 A g^−1^, the GCD curves of NiCo-MOF and NiCo MOF/AB5 confirm their pseudocapacitance characteristics. The *C*_sp_ of NiCo MOF/AB5 was reported to be 744.7, 798.3, 830.1, 844.4, 873.2, 916.1 and 972.2 F g^−1^ at 20, 10, 7, 5, 3, 1 and 0.5 A g^−1^ respectively. Furthermore, the special rate efficiency of NiCo MOF/AB5 was confirmed by the fact that 76.6% of the original specific capacitance was retained even at a rate of 20 A g^−1^. Additionally, an asymmetrical supercapacitor was built employing activated carbon and NiCo MOF/AB5 as the negative electrode and positive electrode, respectively, in which a blank hairy separator is employed, which exhibited an energy density of 33.84 W h kg^−1^ and high power density of 750 W kg^−1^ with a retention of 85.25% of capacitance over 5000 cycles, resulting in its improved stability. Here, the AB present in the NiCo MOF/AB composite works as a conductive network and prevents the aggregation of the NiCo-MOF nanosheets. This nano-design can give a high surface area for the oxidation reaction while also shortening the ion diffusion distances and electron transport path, enhancing electrochemical properties dramatically.^147^ Lee *et al.* prepared a symmetrical supercapacitor based on AC/SIFSIX-3-Ni. Here, SIFSIX-3-Ni (Ni MOF) enhanced the electrochemical properties and acted as a porous conductive element. Additionally, to fabricate a symmetrical supercapacitor, the prepared composites were deposited on an aluminum sheet using the doctor blade technique and then electrolyzed using an ion-gel electrolyte. They prepared a series of samples labeled AC/SIFSIX-3-Ni-*X*, where *X* denotes the mass proportion of Ni-MOF. The specimens were named AC/SIFSIX-3-Ni-1.5, AC/SIFSIX-3-Ni-1.0, AC/SIFSIX3-Ni-0.5, and AC/SIFSIX-3-Ni-0 based on the weight% of the Ni MOF. The electrochemical behavior of AC/SIFSIX-3-Ni electrode at various weight proportions were investigated by CV measurement under the potential window of −0.5 to 1 V. The reaction area of activated carbon may have been filled by the Ni MOF with the increase in the mass balance of Ni MOF to 0.5 wt%, resulting in a higher capacitance value. When the Ni MOF weight percentage was greater than 0.5%, the extra Ni MOF decreased the electrical conductivity, resulting in a lower capacitance value. The Nyquist plot revealed that all the supercapacitors based on the AC/SIFSIX3-Ni nanocomposite produced a semicircle and straight path at higher and lower frequencies, respectively. The electron transport efficiency at the interface of the electrolyte and electrode, which is defined as the charge transfer resistance, is depicted by a semicircle (*R*_ct_). However, for the AC/SIFSIX-3-Ni-0.5 nanocomposite, the *R*_ct_ value was lower than the *R*_ct_ value for other Ni MOF-based supercapacitors. At 20 mV s^−1^, the highest *C*_sp_ of a genuine supercapacitor was determined to be 129 F g^−1^, and a power density of 1940 W kg^−1^ was produced. After 3000 cycles, the manufactured supercapacitor retained 80% of its original capacitance. Here, the Ni MOF might improve electrochemical properties by being introduced into an activated carbon constructed ultracapacitor and functioning as a porous conductive element for the electrode.^148^ Pan *et al.* studies the synthesis of carbon quantum dot (CQD)/Ni-MOF composites *via* a one-pot hydrothermal method for use in energy storage devices. The CQDs were found to effectively enhance the electrical conductivity of Ni-MOF and expose abundant active sites, leading to improved electrochemical performance. The composite material had a specific capacitance of 2457.9 F g^−1^ at 1 A g^−1^ and excellent cycling stability, with 84.5% retention after 5000 cycles. The hybrid supercapacitor using this material as the positive electrode had a wide voltage window of 1.7 V and a high energy density of 67.1 W h kg^−1^ at a power density of 5.1 kW kg^−1^. For high-performance supercapacitors, carbon quantum dots improve the 2D Ni-MOF performance.

### 7.2 1D carbon/2D MOF composite electrode materials for supercapacitors

Wang *et al.* used a simple solvothermal technique to make a NiCo-MOF/MWCNT (flower-string) nanostructure by utilizing a ligand (BPDC). In this study, carboxylated multi-walled carbon nanotubes were employed as a base for producing 2D NiCo-based MOFs *in situ*. Furthermore, the MWCNTs were encapsulated by the 2D NiCo-MOF nanostructure owing to particular connections or interactions between the NiCo-MOF and MWCNTs. They constructed a sequence of NiCo-MOF/MWCNT nanostructures, which they named NiCo-MOF/MWCNT-n. The CV curves of NiCo-MOF and NiCo-MOF/MWCNT composites all at the same scan rate of 25 mV s^−1^ and the galvanostatic charge–discharge profiles of NiCo-MOF/MWCNT and NiCo-MOF composites at 0.5 A g^−1^ current density reveal that NiCo-MOF/MWCNTs-2 possesses the biggest cycle area as well as a lengthy discharge duration confirming its outstanding electrochemical performance. At 0.5 A g^−1^ the specific capacitances of NiCo-MOF/MWCNT-4, NiCo-MOF/MWCNT-3, NiCo-MOF/MWCNT-2, NiCo-MOF/MWCNT-1, NiCo-MOF/MWCNT-0.5, and pure NiCo-MOF have been reported to be 610, 638, 1010, 750, 641, and 526 F g^−1^, respectively. Furthermore, an outstanding rate capability of 760 F g^−1^ even at 10 A g^−1^ was attained, as well as reversibility and good cycling stability with a durability of 828 F g^−1^ (100 percent of the initial capacitance), and at 5 A g^−1^, a coulombic efficiency of approximately 98% was obtained over 3000 cycles. In addition, an asymmetric supercapacitor with a large specific capacitance of 84 F g^−1^ and 88.4% capacitance retention after 3000 GCD cycles was constructed, which displayed good cycling performance. An asymmetric supercapacitor was reported to produce a superior energy density of 19.7 W h kg^−1^ at a power density of 250 W kg^−1^. The composite's remarkable capacitance performance is primarily due to the NiCo-redox MOF's characteristics and MWCNT's high conductivity.^149^

To prepare extremely thin carboxylated carbon nanotube-decorated nickel MOF (Ni-MOF/C-CNTs) nanoplates, Ran *et al.* adopted an *in situ*-induced growth approach. They created a variety of Ni-MOF/C-CNT-X products, with X representing different quantities of C-CNTs. In all the CV measurements, Ni-MOF/C-CNT hybrids and Ni-MOF show a pair of redox peaks with significant potential separations, indicating battery-type characteristics. The specific capacity plot corresponding to current densities indicates that the specific capacities of Ni-MOF/C-CNTs60, Ni-MOF/C-CNTs40, Ni-MOF/C-CNTs20, and Ni-MOF were 581, 680, 524, and 517 C g^−1^ at 1 A g^−1^, and the corresponding retention capacities from 1 to 10 A g^−1^ were 65.6%, 65%, 62.4%, and 57.3%, respectively. Additionally, they constructed a hybrid supercapacitor device (HSC) using activated carbon and Ni-MOF/C-CNTs40 as the negative and positive electrode materials, respectively (Ni-MOF/C-CNTs40//AC). At 1 A g^−1^, a high *C*_sp_ of 97.6 F g^−1^ was produced by the as-prepared HSC device, with a power density of 440 W kg^−1^ and an energy density of 44.4 W h kg^−1^. Moreover, after 3000 sequential charging–discharging cycles, an initial capacitance retention of 77% was obtained. The enhanced capacitive behavior of the decorated architecture could be ascribed to its micro-architecture, which results in abundant electroactive sites and eventually enhances the electrochemical performance.^150^ Sun *et al.* reported the synthesis of a metal–organic framework (MOF)/carbon nanotube (MOF/CNT) composite by a solvothermal method for supercapacitor applications. The corrugated-layered structure of the MOF and the 1D nanotube structure of CNTs were found to provide excellent capacitive performance, with MOF/CNT composites exhibiting higher specific capacitance and rate capacity than the MOF alone. The asymmetric supercapacitor device based on this material also showed high energy density and retention after 5000 cycles. The study suggests that layered MOFs are promising materials for supercapacitor applications and that this synthesis method is an effective way to improve the capacitive performance. MOF(Ni)/CNT composites with a layered structure are suitable for achieving high capacitive performance.

### 7.3 2D carbon/2D MOF composite electrode materials for supercapacitors

Azadfalah *et al.* described the engineering of Co-MOF decorated graphene (CoMG nanocomposite) *via* a one-step *in situ* synthesis method. They prepared different composites, CoMG2.5 and CoMG5, by using a different weight of G (2.5 and 5 mg). A *C*_sp_ of 549.96 F g^−1^ was attained for the composite CoMG5 at a scan rate of 10 mV s^−1^ in a three-electrode system of 6 M KOH. The EIS profile and equivalent circuit profile of the CoM, CoMG2.5, CoMG5, and G electrodes in 6 M KOH solution in a frequency range of 10 mHz to 100 kHz report that CoMG5 possesses the smallest semicircular diameter in the high-frequency zone, indicating better conductivity and reduced charge-transfer resistance. CoM, CoMG2.5, CoMG5, and G electrodes have different *R*_s_ (electrolyte/electrode resistance) values of 3.80, 1.60, 1.01 and 1.13 Ohm respectively. Additionally, an unsymmetrical cell has been built by using CoMG5 as the positive electrode and CA as the negative electrode under a 6 M KOH solution. At a voltage of 1.7 V, the CoMG5//CA asymmetric cell displayed a specific capacity of 50.20 F g^−1^ at 20 mV s^−1^. Moreover, an energy density of 8.1 W h kg^−1^ and a power density of 850 W kg^−1^ at 1 A g^−1^ were also reported. It also demonstrated 78.85% cycling stability over 1000 consecutive cycles. The good capacitance performance of the composite can be ascribed to the greater electrical conductivity of Co-MOF and graphene in the composite, which eventually enhanced capacitive characteristics and smoothened the ion as well as electron movement.^151^ Beka *et al.* reported preparing a two-dimensional nanoplate nickel cobalt-based MOF/reduced graphene oxide heterostructure (2D NiCo-MOF/rGO) as a supercapacitor electrode material. Simple room temperature precipitation is used for the *in situ* growth of NiCo-MOF 2D nanosheets on rGO surfaces. The relative CV curve of the various samples (NiCo-MOF/rGO-3, NiCo-MOF/rGO-2, NiCo-MOF/rGO-1, and NiCo-MOF/rGO-0) provide a comparative understanding of their electrochemical performance. The pseudocapacitive characteristics of the synthesized materials produced from metal redox centers were demonstrated in all samples through the pair of redox peaks. After 5000 cycles of successive charging–discharging, the as-prepared nanocomposite structures demonstrated an exceptional cycling capability of 83.6%. Moreover, at 1 A g^−1^, an excellent *C*_sp_ of 1553 F g^−1^ was also noticed. Additionally, an unsymmetric supercapacitor device was engineered by employing NiCo-MOF/rGO-2 and rGO as positive and negative electrodes respectively (NiCo-MOF/rGO//rGO). At a power density of 3168 W kg^−1^, the asymmetric device displayed an outstanding energy density of 44 W h kg^−1^. Herein the MOF provides a huge surface area with many passages for rapid ion mass movement, while rGO enables quick electron transfer and can improve gross capacitive performance.^152^ Ehrnst *et al.* described an acoustomicrofluidic method that enables the rapid synthesis of homogeneous two-dimensional (2D) metal–organic frameworks (MOFs) and graphene oxide (GO) planar heterostructures for use in electrochemical applications. This method uses intense shear flow and slender-body geometrical confinement to drive *in situ* crystallization and in-plane templating of quasi-2D MOF crystals onto GO sheets. The resulting hybrid material exhibited excellent supercapacitor performance, with a specific capacitance of up to 292 F g^−1^ at a current density of 1 A g^−1^, which is approximately two orders of magnitude larger than that of its 3D MOF/GO counterpart.^153^

MOF-based materials have the potential to be used as capacitive and battery-type electrodes in high-performance supercapacitors. However, many challenges still need to be addressed in order to achieve this goal. These challenges include improving the stability of MOFs in electrolytes under operating potentials, increasing the electrical conductivity of MOFs, and improving the rate capability and exposed surface area of the active surface of MOF-derived metal compounds. Additionally, more research is required on MOF-based materials as electrode materials for HSCs based on organic electrolytes. Overall, the field of MOF-based materials for HSCs is an emerging area that deserves more attention and research effort.

## 8. Black phosphorus (BP)

BP is a p-type semiconductor which has a layered structure as well as a direct and narrow bandgap.^154^ Black phosphorus can exist in different crystal forms. For example, orthorhombic is a form of black phosphorus which is generated by assembling phosphorene sheets by van der Waals force within the layers.^155,156^ There are also other crystal forms of black phosphorus, such as hexagonal and simple cubic.^157,158^ The atomic link in the BP layer had a chair configuration. Black phosphorus, an outstanding 2D material, has the potential to be a next-generation energy storage material. BP has several advantages over other 2D materials such as MXenes and MoS_2_ in terms of supercapacitors and rechargeable batteries: (i) BP has a large volumetric capacitance and high-power density when used as an electrode material in supercapacitors^159^ and (ii) BP also has high charge mobility and outstanding carrier relaxation dynamics when used as an electrode material in supercapacitors. Moreover, black phosphorus shows nonlinear optical properties, good chemical and thermal stabilities, a tunable direct band gap, high theoretical capacity, high charge-carrier mobility, and strong transport anisotropy.^160,161^ Furthermore, it has been discovered that BP exists in four states that can be synthesized: BP nanoparticles, bulk BP, phosphorene, and BP quantum dots (BPQDs).^162^ sp^3^ hybridized P atoms link covalently and produce the unique structure of BP.^163^ The angle formed on the same atomic plane of a layer by two adjacent P atoms is 98.150°, but the angle formed by two adjacent P atoms on opposite planes is 103.690°. P–P bonds on the same and adjacent planes have lengths of 2.164 Å and 2.207 Å, respectively.^164^ The charge/discharge rate and volumetric capacitance of BP nanoflakes are limited due to their low conductivity and the effect of restacking. As a result, carbon materials having great conductivity are employed to optimize the structural integrity and BP flake's conduction.^165–167^ Yang *et al.* utilized a simple sonication process to decorate flexible black phosphorus nanoflakes/CNT nanocomposite paper as a flexible electrode for entirely solid-state ultracapacitors (ASSP). The constructed AASP was discovered to produce the highest power density of 821.62 W cm^−3^, a *C*_sp_ of 41.1 F cm^−3^, and a high energy density of 5.71 mWh/cm^3^. Moreover, even after 11 months, the ASSP system retains a significant volumetric capacitance of 35.7 F cm^−3^ at a scanning speed of 0.005 V s^−1^, suggesting remarkable electrochemical performance and storage stability. Furthermore, it has exceptional mechanical flexibility and cycling stability of 91.5% retention over 10 000 cycles. Here, the highly conductive CNTs serve as active materials, improve the hybrid electrode's conductivity, improve electrolyte shuttling, and prevent BP nanoflakes from stacking again.^165^

**Fig. 10:**
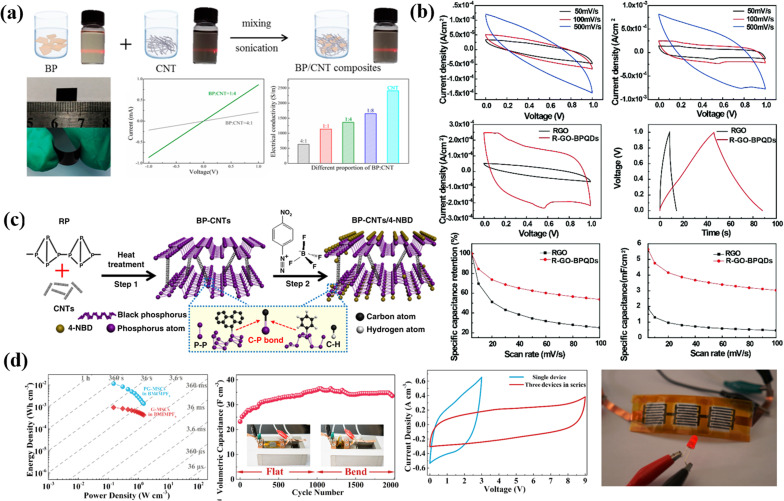
(a) Schematic illustration of the fabrication and characterization of BP/CNT papers, photographs of BP nanoflakes, CNT solution, and mixture dispersion, flexible BP/CNT paper, *I*–*V* curves with mass proportions of 1 : 4 and 4 : 1, and a comparison of electrical conductivity values. (b) Electrochemical performance of supercapacitors based on R-GO-BPQD nanocomposites, showing CV plots at different scan rates, GCD curves at 0.05 mA cm^−2^, specific capacitance, and specific capacitance retention compared between RGO and R-GO-BPQD composite films. (c) Illustration of the synthesis of BP/CNT hybrid by heat treatment of red phosphorous (RP) and CNTs followed by chemical passivation process using 4-nitrobenzene diazonium (4-NBD). (d) Electrochemical performance of PG-MSCs evaluated through Ragone plot comparison with G-MSCs, cycling stability under flat and bending states, CV curves of single and serial PG-MSCs, and powering a light-emitting diode (LED) with three serial PG-MSCs.

Wu *et al.* create a black phosphorus hybrid-structured compound that is chemically linked with carbon nanotubes. The as-prepared BP/CNT hybrid is then combined into non-woven fibre textiles *via* a microfluidic spinning process. However, the hybrid nanoarchitectured device produced a very high energy density of 96.5 mW h cm^−3^ with an excellent volumetric capacitance of 308.7 F cm^−3^. Moreover, a good retention of 90.2% of original capacitance was observed over 10 000 cycles, showing its outstanding stability. This demonstrated that all the electrochemical behavior is superior to that of capacitors based only on carbon nanotubes or black phosphorus. The wide 2D architecture, chemical linking between CNTs and BP, and favourable path channels inside the network, all worked together to increase flooding and ion transport, resulting in better capacitive behavior.^166^ Fu *et al.* developed a straightforward laser fabrication of flexible planar supercapacitors composed of black phosphorus quantum dots (BPQDs) and GO. The interdigital pattern on the composite films was created using DLW technology. They made planar all-solid-state supercapacitors by employing R-GO-BPQD composite electrodes to measure the electrochemical characteristics of the as-prepared composite film. RGO-based supercapacitors, on the other hand, were also built. The R-GO-BPQD electrode-based supercapacitor has a more rectangular form and a larger integral area at 100 mV s^−1^, indicating that the charge capacity of the R-GO-BPQD composite constructed ultracapacitor is greater than that of the RGO constructed ultracapacitor. At a scanning speed of 5 mV s^−1^, a *C*_sp_ of 5.63 mF cm^−2^ was observed for the as-prepared R-GO-BPQD constructed ultracapacitor. Moreover, when the scan rate was changed from 5 mV s^−1^ to 100 mV s^−1^, a *C*_sp_ retention of 53.75% for the R-GO-BPQD composite constructed ultracapacitor was noticed. The capacitance retention remains as high as 98.09% after 1000 bending cycles, exhibiting the exceptional flexibility and outstanding electrochemical stability of the material. Moreover, they prepared an integrated device (three R-GO-BPQD-based supercapacitors in series and three in parallel) which provided a reasonable performance of 3 V of voltage extension, which can readily power an LED. The synthesized supercapacitors demonstrated higher specific capacity and higher rate performance than those based on a single RGO electrode due to the synergistic effect of abundant active sites at the surface of BPQDs and high conductivity obtained from reduced GO (RGO).^168^Fig. 10 shows the fabrication and characterization of BP/CNT papers (Fig. 10a), and the electrochemical performance of supercapacitors based on the R-GO-BPQD nanocomposites (Fig. 10b) and PG-MSCs (Fig. 10c). The BP/CNT paper characterization and fabrication shown in schematic illustration, photos of BP nanoflakes, CNTs solution, mixture dispersion, flexible BP/CNT paper, *I*–*V* curves, and a comparison of electrical conductivity values at different mass proportions. The electrochemical performance of the supercapacitors measured by CV plots at different scan rates, GCD curves at 0.05 mA cm^−2^, specific capacitance, and retention compared to RGO films. The electrochemical performance of the PG-MSCs was evaluated through a Ragone plot comparison, cycling stability under flat and bending states, CV curves of single and serial PG-MSCs, and powering an LED with three serial PG-MSCs. Using a simple one-step filtration process and an electrolyte (BMIMPF6), Xiao and colleagues created a nanoarchitecture based on black phosphorus/graphene for micro-supercapacitors (PG-MSCs). The PG-MSCs have a remarkable capacitance performance of 11.6 mWh/cm^3^ energy density and 37.0 F cm^−3^ volumetric capacitance, and 9.8 mF cm^−2^ areal capacitance. Moreover, the PG-MSCs deliver a superior volumetric capacitance of 2.42 F cm^−3^ even at 1000 mV s^−1^. Herein the high conductivity graphene can act as a high-speed electron transfer pathway and mechanical framework, allowing phosphorene nanosheets to be used for energy storage more efficiently.^169^ Sharma *et al.* described the synthesis of vanadium disulfide-black phosphorus (VS_2_-BP) hybrids by a one-pot hydrothermal-assisted method for supercapacitor applications. The study found that the charge storage kinetics of the best-performing VS_2_-BP hybrid demonstrated a dominance of the pseudocapacitive nature of the material. Furthermore, an all-solid-state symmetric device was developed with the highest specific areal capacitance of 203.25 mF cm^−2^ and maximum areal energy density of 28.22 μW h cm^−2^. The study also found that the improved density of states in the VS_2_-BP hybrid is due to the charge accumulation region between VS_2_ and BP monolayers and the charge transfer.^170^

BP is an emerging and promising 2D material that has received broad interest in various applications, such as flexible supercapacitors (FSCs), owing to its unique structure and intrinsic electrochemical properties. In this paper, we have reviewed BP-based FSCs based on the structure and properties of BP for the fabrication and final configurations of FSCs. The quality of BP nanostructures is a dominant factor related to the electrochemical performance of supercapacitors, which greatly influences the mechanical properties of BP-based flexible electrodes. Therefore, several challenges must be addressed to design a high-performance BP-based supercapacitor with customizable flexibility and stretchability. They include thickness-controllable and scalable production of high-quality 2D BP, stability of 2D BP in an ambient environment, and higher energy and power density of supercapacitors. Research in these areas is essential to achieve satisfactory energy storage performance and meet the demands of practical applications of supercapacitor devices.

## 9. Perovskites

Perovskites are a group of materials possessing the general formula ABX_3_ and have a structure similar to the CaTiO_3_ crystal prototype. The anion “X” is present on the unit cell's faces while the corner position of the unit cell is occupied by the cation “A” and the centre position is covered by the cation “B”. Gustav Rose, a German mineralogist in 1839, identified calcium titanate, a crystalline structure known as perovskite, or CaTiO_3_, and later by a Russian mineralogist, the material was named by Lev Perovskite. Following that, any compounds with the crystalline structure the same as CaTiO_3_ were named perovskite.^171–173^ In addition, a big monovalent cation is considered as “A” that has a cubic-like structure and covers the cuboctahedral sites, while the octahedral site is occupied by the small divalent metal cation “B” and “X” is a halogen anion. Oxygen, nitrogen, or carbon can alternatively be used as the X element. A should be a divalent cation, and B should be a tetravalent cation when the anion of perovskite is an oxide. Like that, monovalent cations are considered as A sites, and divalent cations are B sites when the anion of the perovskite structure has a halogen.^174,175^ There have been a variety of perovskite oxide derivatives reported so far, such as Ruddlesden–Popper perovskite oxides ((AX)(ABX_3_)_*n*_)^176^ and double perovskite oxides (AABBX6), where A and A: alkaline earth metals or lanthanides, and B and B: transition elements.^177^ The dielectric (Ba,Sr)TiO_3_, the ferroelectric BaTiO_3_ and PbTiO_3_, the electro-strictive Pb(Mg,Nb)O_3_, the multiferroic BiFeO_3_, the piezoelectric Pb(Zr,Ti)O_3_, and the magneto-resistant (La,Ca)MnO_3_ are some examples of typical oxides.^178^ Metal halides are classified into two classes based on the type of cation: entirely inorganic and hybrid organic–inorganic metal halides. The cation A represents a monovalent alkali metal (such as K and Cs) in the first case and a tiny organic cation (like CH_3_NH_3_) in the second case.^179,180^ Because of their superior properties like electrochemical stability, thermal stability, and tunable structure, perovskite materials have drawn a lot of interest as energy storage materials. Cao *et al.* described the decoration of an activated carbon/CaTiO_3_ perovskite composite (Ti-1-CAC) to improve the capacitance performance of activated carbon without involving any conductive agent. Ti-1-CAC showed a much larger capacitance than any other electrode and was found to exhibit a capacitance of 270 F g^−1^ for a three-electrode cell. A *C*_sp_ of 185 F g^−1^ was also observed for a two-electrode system at 0.5 A current density in 6 M KOH. The as-prepared composite exhibited a capacitance retention ratio of ∼85% at a current density of 20 A g^−1^. They also discovered that Ti-1-CAC has better charge–discharge reversibility and electrical double-layer characteristics than other materials. Additionally, they noticed that with a gradual increase in power densities from 375 to 5065 W kg^−1^, the energy density reduces marginally from 26.3 to 22.5 W h kg^−1^ for Ti-1-CAC. The inclusion of CaTiO_3_ in carbon pores could increase the electrochemical properties of activated carbon for use in supercapacitors by offering unique electronic features on carbon/CaTiO_3_ as well as pore systems.^181^ Gonzalez *et al.* used a spin-coating process to coat carbon nanotubes with Ca_2.9_Nd_0.1_Co_4_O_9+*δ*_ (CaNCo) with a weight ratio of (2 : 98) CaNCo perovskite to CNTs to create a solid-state ultracapacitor. They used the following topologies to study two CNT-based SCs: CNT + CaNCo/SAM/CNT as the CaNCo system and CNT/SAM/CNT as the CN system. CNT/CaNCo was employed as the anode for all systems, and the cathode was an electrode constructed entirely of CNTs. They found that the discharge time of the CaNCo device was 8.6 seconds longer than that of the CN device. The CV measurements were carried out at scan rates of 50, 70, and 100 mV s^−1^ for the CN device. There were no oxidation or reduction peaks for any scan rate, which shows that the devices stored charge *via* the EDL process. The CV curve undergoes significant modifications when the CaNCo perovskite is introduced. The current of the CaNCo perovskite was first increased by ∼288%. Second, as shown by the green circles, some faradaic peaks occurred, which indicates that the energy storage mechanism switched from the EDL to redox processes. As a consequence, the CN system had a *C*_sp_ of 53.6 F g^−1^ with an energy density of 16.7 W h kg^−1^, whereas the CaNCo system produced a capacitance value of 620.4 F g^−1^ and an energy density of 95 W h kg^−1^ at 1 A g^−1^.^182^

Galag *et al.* reported that mixing SrRuO_3_ and highly conductive graphene sheets can enhance the specific capacitance of perovskite materials. Here, the SrRuO_3_-RGO composite (SRGO) was prepared by mixing SrRuO_3_ with RGO, followed by ultrasonication. In addition, by altering the mass percentages (25 to 80% w/w) of SrRuO_3_ and RGO and the composition of the electrolytes, they reported the capacitive properties of the resulting SRGO composite. The CV curves of SRGO-50, SRO, SRGO-80, and RGO obtained at a scan rate in 1.0 M NaNO_3_. SRGO-50, RGO, and SRGO-50 were all rectangular; however, SRO has two redox peaks, which are associated with its pseudocapacitance. A capacitance value of 214.8 F g^−1^ for SRGO-80 and 220.4 F g^−1^ for SRGO-50 has been recorded at a 2.0 mV s^−1^ scan rate. SRGO-50, on the other hand, at a comparatively increased current density at 25.0 A g^−1^, demonstrated 56.5% degradation at rapid charge–discharge currents. Moreover, SRGO-50 retained 93.3% of its original capacitance over 1000 cycles. Furthermore, they discovered that at a current density of 1 A g^−1^, the involvement of RGO enhanced the capacitance of SRO from 24.5–101 F g^−1^ (at 1.0 M H_3_PO_4_), 13.8–62.4 F g^−1^ (at 1.0 M NaNO_3_), and 52.4–160 F g^−1^ (at 1.0 M KOH). The EIS results show that combining SRO with RGO lowers the solution and charge transfer resistances. Here, the combination of graphene with perovskites improves the specific capacitance of graphene by introducing pseudocapacitance as well as the double-layer capacitance of graphene sheets. Perovskites also act as spacers between graphene plates, preventing them from aggregating. The combination of the fast-faradaic redox activity of Ru ions from SRO with the high conductive and vast surface area of graphene sheets causes this synergetic characteristic between RGO and SRO.^183^ Tomar *et al.* described the synthesis of strontium titanate (STO) with a cubic structure using a sol–gel method. The STO sample was uniformly coated on a 1 cm^2^ graphite sheet (SrTiO_3_/graphite sheet). In this study, STO electrodes were studied for both all-solid-state symmetric supercapacitor devices and aqueous symmetric supercapacitor devices. At 0.63 A g^−1^, an excellent specific capacitance of 212.5 F g^−1^, as well as an areal capacitance of 85 mF cm^−2^, was observed for the aqueous STO//STO supercapacitor cell, along with a capacitance retention of as high as 74.5% at 2.5 A g^−1^ and 42.85% at 5 A g^−1^. It also had the highest energy density of 27.8 W h kg^−1^ and power density of 1921 W kg^−1^. Moreover, after consecutive 5000 GCD cycles, only a 1% loss in cycling stability was reported. The CV test of STO thin-film electrodes at various scan rates indicates that charge is stored *via* a pseudocapacitive mechanism. In addition, a solid-state symmetric cell was built, in which PVA/KOH gel was employed as the electrolyte, with had a maximum energy density of 3.62 W h kg^−1^ and a power density of 965 W kg^−1^. Furthermore, after 3000 GCD cycles, the cycling stability test of a solid-state STO//STO cell at 1 A g^−1^ showed a capacitance retention of 71.6%.^172^ Recently Shafi *et al.* examined the effects of cation substitution on the symmetry and structural stability of ABO_3_-type perovskite oxides, which are materials with potential for use in energy storage devices. The researchers found that substituting La^3+^ with Sr^2+^ and Mn^3+^ with Fe^3+^ improved the crystal symmetry and mixed ionic-electronic conductivity of the perovskite. They also found that using exohedral carbon nano-onions as a negative electrode in a hybrid supercapacitor design improved the rate capability, resulting in a high-rate hybrid device with good energy density.^184^

Perovskite oxides have recently been widely employed as electrode materials for supercapacitors owing to their unique structures, compositional flexibilities, and inherent oxygen vacancies. The use of perovskite oxides as active materials for intercalation-type capacitors is characterized by high oxygen vacancy concentrations and does not require a high surface area to achieve a high energy storage capacity. Despite many efforts in this field, several aspects still need to be considered in the design of perovskite oxide electrode materials for SCs. One of the challenges is to increase the valence-state change of the B-site cation with a low energy barrier in perovskite oxides. Another challenge is to dope non-metal at the B site of ABX_3_, which can improve the phase stability, electrical conductivity, and oxygen vacancies of perovskite oxides. Additionally, more research is required on RP perovskite oxides with higher *n* values as advanced SC electrode materials. Another challenge is to find a way to control the morphology of perovskite oxides to improve their electrochemical performance by increasing their surface area and exposing more active sites. Finally, the use of redox electrolytes may be promising for boosting the electrochemical performance of SCs.

## 10. Conclusion and future prospects

Electrochemical conversion and energy storage devices, such as fuel cells, batteries, and electrochemical capacitors (ECs), have been used in recent years. However, their unique characteristics, such as very high capacitance, high power density, high-speed charge/discharge, and long cycle life supercapacitors (SCs), have attracted much attention in recent years as electrochemical energy storage devices. The different dimensional carbon materials used in supercapacitors have enhanced their capacity. The different types of pores present in carbon materials facilitate the penetration of ions inside the bulk. In summary, recent achievements in the development of metal oxides, mixed transition metal oxides, MXenes, transition metal dichalcogenides (TMDs), metal hydroxides, graphitic carbon nitrides (g-C_3_N_4_), black phosphorus, layered double hydroxides (LDHs), metal–organic frameworks (MOFs), and perovskite nanoarchitectures as supercapacitor electrode materials have been studied. In contrast to pure carbon-related supercapacitors, the capacitive properties are limited by the surface area, energy density, and pore size. Owing to the availability of mesoporous structures, pseudocapacitive nature, low toxicity, good stability, cost-effectiveness, and abundant accessibility, metal oxides, mixed metal oxides, metal hydroxides, TMDs, MXenes, LDHs, g-C_3_N_4_, MOFs, black phosphorus, and perovskites have been employed as supercapacitor electrode materials to attain improved capacitance properties.

Supercapacitors are important components in various applications such as hybrid vehicles, communication devices, military warheads, laser technology, mobile phones, solar cell energy storage, and uninterruptible power supplies. Supercapacitors have gained much interest because of several significant aspects, including rapid charging–discharging procedures, high power densities, and outstanding cycling stability. Supercapacitors are currently employed in commercial applications; however, their low energy density and high cost prevent them from replacing batteries. Therefore, SCs must solve two major challenges: lower energy density and high cost, while sacrificing their rate performance and high cycle life. Many efforts have recently been directed toward creating nano-architecture electrode materials with improved operating voltages and outstanding specific capacitances, and to solve the technical challenges associated with poor energy density. Two approaches have been explored for high-voltage SC designs: substituting organic electrolytes or aqueous electrolytes with ILs with a wide potential area and developing hybrid asymmetric SCs that use both faradaic (as a source of energy and power) and capacitive electrodes. Flexible solid-state supercapacitors are another technology used to create high power densities. Solid-state supercapacitors are potential options for energy storage systems because of their ease of integration, flexibility, high power density, and safety. Higher operating voltages can help save money in terms of energy bills. The energy density increases as the working potential window widens, requiring fewer electrolytes, separators, and materials to store the same amount of energy. SC electrode materials have sparked much interest in recent years as a way to make energy-storage systems that are both well-performing and cost-effective. Waste materials are a cost-effective way to fabricate supercapacitor electrodes. However, they still need a lot of work (fabrication methods, electrolyte selection, *etc.*) before they can be sold as raw electrode materials for trading purposes.

The development of novel electrode materials is critical to meet the urgent demand for high-performance energy storage and conversion devices. Furthermore, various research groups have increased their efforts to improve the overall performance of supercapacitors using carbon materials with different dimensions (0D to 3D) as well as pseudocapacitive materials for supercapacitors, among other techniques. The insertion of different-dimensional carbon nanomaterials into ASC devices resulted in improved electrochemical performance. This review comprehensively summarizes the recent progress in carbon-based hybrid networks with 2D electrode material-based supercapacitors. From the available representative results and comparisons, we draw conclusions and define the scope of future research.

1. There is still room for improvement in the cycling behavior retention and power density of all 2D material pseudocapacitors because the stagnant redox kinetics associated with inherent ion/electron transport severely restricts their performance. Many strategies have been successfully employed to overcome this limitation, including doping, frameworks, core–shell architectures, rigid hierarchies, porous structure architectures, and multi-metal centers. However, there is still a long way to go before ASC devices can be designed from such materials for long-term practical applications.

2. The large surface area, excellent electrical conductivity, and processability are characteristics of carbon nanomaterials, which make them potential candidates for energy storage. Owing to their interconnected porous structure, large interstitial surface area, mass advantage, and ability to provide suitable charge pathways, 3D carbon channels are preferred.

3. Carbon-2D material networks have shown excellent electrochemical performance and have emerged as viable supercapacitor candidates. Carbon networks and pseudocapacitive materials were combined in these hybrid nanomaterials to increase the energy density and cycling performance of the individual components.

4. The carbon-2D electrode material network helps to attain a high operating potential window by synchronizing their different charge storage mechanisms to reach practical, real-world applications.

5. The hybrid composite electrode with 3D structures exhibited the best potential in terms of overall electrochemical performance. Hybrid supercapacitors can provide increased power, energy, and lifespan as an alternative to batteries.

However, drawbacks still prevent hybrid supercapacitors from being widely used in future energy-storage devices. They include (i) mass ratio optimization, (ii) synthesis technique optimization to avoid restacking towards agglomeration, (iv) compatible homogeneous integrity, and (v) further study of negative electrodes to understand the wide potential window need are some aspects that need to be further optimized for better performance.

The advantages of carbon-based 2D network SCs for energy storage systems include ease of integration, flexibility, high power density, and safety. The energy costs can be reduced by using higher operating voltages. Fewer electrolytes, separators, and other materials are required to store the same amount of energy when the working potential window increases. Energy storage systems that are both cost-effective and high-performing have recently attracted the attention of researchers who use SC electrode materials. For this purpose, supercapacitor electrodes can be made from waste materials. There is still a lot of work to be done before they can be sold as raw electrode materials for trading purposes (fabrication methods, electrolyte selection, *etc.*).

## Author contributions

P. K. S., and C. P. L. conceived and planned the work and edited the manuscript. N. K., D. K. and S. G. contributed to the writing.

## Conflicts of interest

There are no conflicts to declare.

## Supplementary Material
